# Supplementary material for: Mechanical strain focusing at topological defect sites in regenerating Hydra
Source: Development. 2025 Mar 3;152(4):DEV204514. doi: 10.1242/dev.204514 (PMC11925399; doi:10.1242/dev.204514)
Supplement: Supplementary information [file develop-152-204514-s1.pdf]

Supplementary Materials and Methods
Mechanical strain focusing at topological defect sites in regenerating Hydra

Yonit Maroudas-Sacks,[1] S. Suganthan,[2] Liora Garion,[1] Yael Ascoli-Abbina,[1] Ariel Westfried,[1] Noam Dori,[1] Iris Pasvinter,[1] Marko Popović,[2,3,4] and Kinneret Keren[1,5]

[1]Department of Physics, Technion-Israel Institute of Technology, Haifa 32000, Israel
[2]Max-Planck Institute for Physics of Complex Systems, MPI-PKS, Nothnitzer Str. 38, Dresden, 01187, Germany
[3]Center for Systems Biology Dresden, Pfotenhauerstrasse 108, 01307 Dresden, Germany
[4]Cluster of Excellence Physics of Life, TU Dresden, Dresden, Germany
[5]Network Biology Research Laboratories and Russell Berrie Nanotechnology Institute, Technion–Israel Institute of Technology, Haifa 32000, Israel

I. MODEL OF REGENERATING HYDRA

Here we describe the vertex model of a regenerating Hydra that we developed to study the dynamics of Hydra tissue during the early stages of regeneration. Our model describes a tissue spheroid that encloses an incompressible fluid-filled lumen. The mechanics of the tissue is defined by an energy function that accounts for tissue elasticity. Furthermore, our model contains a nematic tensor field and a scalar morphogen field that are coarse-grained on the scale of individual cells and represented by a nematic tensor and the number of morphogen molecules in each cell. We first employ this model to study how active stresses generated by the nematic field lead to focusing of area strain at locations of nematic defects. Then, we incorporate a positive feedback-loop between tissue mechanics, nematic defects and a diffusing morphogen and show that it can provide a robust mechanism for +1 nematic defect stabilisation, co-localised with a morphogen peak.

A. Vertex model of Hydra body mechanics

We model the mechanics of regenerating Hydra tissues using a custom vertex model of a curved deformable surface. We represent the Hydra myoepithelial bi-layer by a single layer of cells that encloses a lumen. Cells are defined by a closed sequence of straight line segments, called cell bonds. Each bond is shared by two neighboring cells and its end-points are called vertices. Vertices, at which three or more bonds meet, are the degrees of freedom defining the state of the cellular network (Fig. S9A). The cellular network topology is defined by cell neighborship relations, which can change by cell rearrangements, also called T1 transitions [1].

In our model, cell bonds do not yet uniquely define a cell surface, as all the bonds of a given cell will in general not lie in the same plane (Fig. S9A). To define the surface of a cell c we first define its center to be the mean position of all its vertices i

$$\vec{R}_c = \frac{1}{M_c}\sum_{i\in c}\vec{r}_i, \tag{1}$$

where M_c is the number of cell vertices. We define the cell surface as a set of triangles generated by connecting each cell bond with the cell center by straight line segments. For each such triangle we define the triangle normal vector,

$$\hat{n}_{c,i} = \frac{(\vec{r}_i - \vec{R}_c)\times(\vec{r}_{i+1} - \vec{R}_c)}{\left|(\vec{r}_i - \vec{R}_c)\times(\vec{r}_{i+1} - \vec{R}_c)\right|}, \tag{2}$$

where the vertices i and $i+1$ are assigned such that the normal vector points outward, relative to the enclosed lumen. We use this convention in the rest of this Supplemental Material. The cell area is calculated as the sum of the triangle areas,

$$A_c = \frac{1}{2}\sum_{i\in c}\left|(\vec{r}_i - \vec{R}_c)\times(\vec{r}_{i+1} - \vec{R}_c)\right| \quad . \tag{3}$$

We define a tangent plane for each cell by calculating a cell normal vector $\hat{n}_c$ which corresponds to the area weighted average of the normal vectors of all the cell triangles. In each cell c, we define a cell coordinate system $\mathbf{O}_c$ centered at the cell center $\vec{R}_c$, and the $z-$axis of the coordinate system coincides with the cell normal vector $\hat{n}_c$.

The volume of the lumen enclosed by the cellular network is evaluated as the sum of tetrahedral volume elements,

$$V_{\text{lumen}} = \frac{1}{6}\sum_{c\in\text{cells}}\sum_{i\in c}\left[(\vec{r}_i - \vec{R}_c)\times(\vec{r}_{i+1} - \vec{R}_c)\right]\cdot\vec{r}_i \quad . \tag{4}$$

We define the mechanical energy of the cellular network by incorporating standard vertex model terms; cell area elasticity, bond tension, bond perimeter elasticity and an additional term that introduces a bending energy to reflect the energetic cost of bending a tissue of finite thickness,

Development • Supplementary information

$$E_0(\{\vec{r}_v\}) = \sum_{c\in\text{cells}} \frac{K_c}{2}(A_c - A_{c,0})^2 + \sum_{b\in\text{bonds}} \Lambda_b L_b + \sum_{c\in\text{cells}} \frac{\Gamma_c}{2} P_c^2 + \sum_{b\in\text{bonds}} \beta_b L_b(1 - \hat{n}_{b,c}\cdot\hat{n}_{b,c'}) \quad .$$

Here, K_c and $A_{c,0}$ are the area elastic modulus and preferred cell area, Λ_b and L_b are bond tension and length of bond b, Γ_c and P_c are perimeter elastic constant and perimeter of cell c, β_b is the bending elastic modulus and $\hat{n}_{b,c}$, $\hat{n}_{b,c'}$ are, respectively, the normal vectors of cells c, c' that share the bond b and their product can be expressed as $\hat{n}_{b,c}\cdot\hat{n}_{b,c'} = \cos\theta_b$, where θ_b is the angle between the normal vectors. In the simulations of Hydra regeneration we use a constant value (i.e. a cell or bond independent value) for the following parameters: $K_c = K$, $A_{c,0} = A_0$, $\Lambda_c = \Lambda$, $\Gamma_c = \Gamma$, and $\beta_b = \beta$. For the numerical values of all the parameters used in the simulations see Table 1.

We further need to account for the fact that the fluid enclosed by the cellular network is incompressible and therefore its volume V cannot be changed by the mechanical forces exerted by the cellular network. To implement this volume constraint we introduce a Lagrange multiplier μ. Note that this method allows us to change V in time to represent the changing lumen volume due to osmotic fluid influx and occasional fluid eflux following tissue ruptures. However, for simplicity, in this work we keep V constant over time. The mechanical energy function becomes,

$$E(\{\vec{r}_v\}) = E_0(\{\vec{r}_v\}) + \mu V(\{\vec{r}_v\}). \tag{5}$$

The energy gradient generates a force on each vertex

$$\vec{F}_v = -\frac{\partial E(\{\vec{r}_v\})}{\partial \vec{r}_v}, \tag{6}$$

which we evaluate analytically for each of the terms in the energy function $E(\{\vec{r}_v\})$.

In order to describe the nematic pattern of actin fibers we introduce a nematic field on the surface of the cellular network represented in a coarse-grained manner by assigning a two-dimensional nematic tensor $\boldsymbol{q}_c$ to each cell. The nematic tensor $\boldsymbol{q}_c$ lies in the cell tangent plane.

The myoepithelial actin fibers exert mechanical stress upon activation [2]. In our model we introduce a nematic active stress in each cell which is proportional to the average cell nematic tensor in the cell and its neighbors $\boldsymbol{\sigma}_c^a = -\zeta_c|\langle\boldsymbol{q}\rangle_c|\boldsymbol{q}_c$. The activity parameter ζ_c reflects the activation level of cell c, and can vary from cell to cell depending on the activation pattern in the tissue. Here, $|\langle\boldsymbol{q}\rangle_c|$ is the magnitude of the nematic tensor obtained as the average of the cell c nematic and projected nematics of neighboring cells to the tangent plane of cell c (Fig. S9C). In this way the norm of the active stress is reduced when nematics in neigboring cells are not fully aligned.

We translate the active stress generated by each cell c to the corresponding forces on the vertices $\vec{f}^a_{c,v}$ of that cell using the active stress scheme from Ref. [3], generalized to three dimensions. The total active force on each vertex is the sum of the active forces from each of its cells $\vec{f}^a_v = \sum_{c(v)} \vec{f}^a_{c,v}$.

The dynamics of the cellular network follows the overdamped dynamical equation for the vertex positions,

$$\gamma\frac{d\vec{r}_v}{dt} = \vec{F}_v + \vec{f}^a_v \tag{7}$$

where γ is a dissipation coefficient. In addition to movement of vertices, changes in the state of the cellular network can also occur through cell rearrangements during which cells exchange neighborship relations. In the vertex model such changes correspond to T1 transitions during which a bond between cells is lost and/or created. In our model, we implement T1 transitions so that whenever a bond becomes smaller than a threshold value $\epsilon_{T_1} = 0.005\sqrt{A_0}$, the bond is removed and its two vertices are merged into a single vertex. For example, merging of two three-fold vertices gives rise to a single four-fold vertex. If at any time a multi-fold (four or higher) vertex exists in the network we perform a check to test whether a new bond is energetically favorable, compared to retaining the multi-fold vertex. In particular, we consider all possible topological splittings of the multi-fold vertex into two vertices i and j, while keeping the length of the new bond at zero. For each of these topologies (i, j), we calculate the force acting to extend the bond length: $f_{ij} = (1/2)|\vec{F}_i + \vec{f}^a_i - \vec{F}_j - \vec{f}^a_j| - (\Lambda_b + \Gamma P_{c(i)} + \Gamma P_{c(j)})$ where i and j are the two vertices of the new bond, $\vec{F}_i$ and $\vec{f}^a_i$ are the forces acting on vertex i as described above, and $c(i)$ and $c(j)$ are the two cells that share the newly opened bond. We find the maximal value of f_{ij} among all possible splittings (i, j) and, if it is positive, we generate that bond. Then, we set the length of the new bond to $l_0 = 10\epsilon_{T1}$ along the axis of the force difference $\vec{F}_i + \vec{f}^a_i - \vec{F}_j - \vec{f}^a_j$. If the maximal f is negative, we retain the multi-fold vertex.

B. Morphogen field

We introduce a morphogen field to our model of Hydra. The morphogen field is thought to describe an activator associated with the formation of the head organiser region [4]. We define the number of morphogen molecules N_c in each cell c. The dynamics of the morphogen field is specified by a production-diffusion-degradation equation

Development • Supplementary information

as follows,

$$\frac{dN_c}{dt} = D\sum_{c'} L_{b(c,c')} h_{b(c,c')} \left(\frac{N_{c'}}{V_{c'}} - \frac{N_c}{V_c}\right) - r_- N_c + r_+ f(\langle\epsilon\rangle_c), \quad (8)$$

where the first term corresponds to diffusive fluxes of morphogen $J_{c'\to c}$ from neighboring cells c' to the cell c (Fig. S9B), $L_{b(c,c')}$ and $h_{b(c,c')}$ are bond length and lateral interface height shared by cells c and c', and D is the transport coefficient for morphogen diffusion between neighboring cells. r_- is the degradation rate and r_+ is the production rate amplitude and $f(\langle\epsilon\rangle_c)$ is a sigmoid function that describes the morphogen production as a function of the cell area strain $\epsilon_c = \ln\left(A_c/A_c^{\text{ref}}\right)$, where $A_c^{\text{ref}} = A_c(t=0)$ is the reference area, chosen as the area of cell c at the start of the simulation, and $\langle...\rangle_c$ indicates average over cell c and its neighbors. Specifically, we use $f(\epsilon_c) = \frac{1}{2}\left(\tanh(w(\langle\epsilon_c\rangle_c - \epsilon_{th})/\epsilon_{th}) + 1\right)$ where ϵ_{th} is the strain threshold to produce morphogen and the parameter w sets the range of strains over which the production is activated with increasing strain. In this work we use $w = 10$. Furthermore, we consider that diffusion inside cells is much faster than across cell boundaries so that the concentration inside a cell is approximately constant, compared to gradients across cell boundaries. Since the cell volume is not easily changed by mechanical deformation of cells we simplify Eq. 8 by introducing the 2D concentration $\phi_c = N_c/A_c$ so that our morphogen dynamics equation becomes

$$\frac{dN_c}{dt} = D\sum_{c'} L_{b(c,c')} (\phi_{c'} - \phi_c) - r_- N_c + r_+ f(\langle\epsilon\rangle_c) \quad (9)$$

where we have used the approximation that the height of cell-cell interfaces does not vary significantly across a single cell so that we can define a cell height h_c which satisfies $A_c h_c = V_c$.

C. Dynamics of the nematic field

The evolution of the nematic in each cell is given by

$$\frac{d\boldsymbol{q}_c}{dt} = \frac{1}{\tau_q}\langle\boldsymbol{q}\rangle_c + \alpha(\vec{\nabla}\phi_c \otimes \vec{\nabla}\phi_c)^{ST} + m(\boldsymbol{q}_c)\boldsymbol{q}_c, \quad (10)$$

where the first term is the standard alignment to the neighbor nematic orientation with a rate $1/\tau_q$. In the continuum limit on flat surfaces this term would correspond to the lowest term in the long-wavelength expansion of the nematic interaction energy in the one-constant approximation [5]. Here, we adjust the coupling to the curved surface by defining a neighbor-averaged nematic alignment as $\langle\boldsymbol{q}\rangle_c = \sum_{c'\in c} \boldsymbol{P}_c(\boldsymbol{q}_{c'})/M_c$ where the cells c' are neighboring cell c, $\boldsymbol{P}_c(\boldsymbol{q}_{c'})$ is the projection of the nematic in cell c' to the tangent plane of cell c (Fig. S9C), and M_c is the number of neighbors of cell c. The second term in Eq. 10 corresponds to nematic-morphogen interaction that favors alignment of the nematic to gradients of the morphogen field, with a coupling coefficient α. Here the superscript ST denotes the symmetric traceless part of the tensor, and $\vec{\nabla}\phi_c$ denotes the discrete gradient of the morphogen field at $\vec{r}_c$, determined as described below (Fig. S9D). The last term in Eq. 10 imposes the magnitude constraint $|\boldsymbol{q}_c|^2 = 1$.

We calculate the discrete gradient $\vec{\nabla}\phi_c$ of the morphogen field following the standard procedure based on least square optimization as follows. We define a cost function,

$$e_c = \sum_{c'} \left[\phi_{c'} - \phi_c - \vec{\nabla}\phi_c \cdot \vec{r}_{c,c'}\right]^2 \quad (11)$$

where the sum is over cells c' that are neighbors of cell c. To find the distance between neighboring cell centers we deterime the geodesic line connecting them along the surface (determined by the cell triangles), see Fig. S9D. Concretely, we define the displacement vector $\vec{r}_{c,c'}$ between neighboring cell centers by rotating each neighboring cell c' of the cell c around their shared bond $b(c,c')$ such that its center lies in the tangent plane of the cell c. The length of $\vec{r}_{c,c'}$ is equal to the distance between cell centers along the geodesic.

The discrete gradient $\vec{\nabla}\phi_c$ is then defined as the one that minimizes this cost function and is given by

$$\vec{\nabla}\phi_c = \boldsymbol{M}_c^{-1} \cdot \sum_{c'} (\phi_{c'} - \phi_c)\vec{r}_{c,c'}, \quad (12)$$

where

$$\boldsymbol{M}_c = \sum_{c'} \vec{r}_{c,c'} \otimes \vec{r}_{c,c'}. \quad (13)$$

1. *Transport of the nematic*

As the cells move in space, the nematic $\boldsymbol{q}_c$ in each cell is convected and co-rotated with the cell. Nematic convection is implemented by maintaining the nematic position at the cell center as it moves. To implement the co-rotation of the nematic we devised a two step algorithm that accounts for out-of-plane rotations, as defined by changes of the cell normal vector, and then the remaining in-plane rotations of each cell: In the first step we determine the cell normal vector $\hat{n}_c$ at times t and $t+\Delta t$, see Eq. 2. To make sure that the nematic remains in the cell tangent plane at the time $t+\Delta t$, we rotate the nematic by a rotation matrix $\boldsymbol{R}_1$ that corresponds to rotation between the two normal vectors: $\hat{n}_c(t+\Delta t) = \boldsymbol{R}_1\hat{n}_c(t)$. In the second step we account for rotation of the cell within its tangent plane defined by the normal vector $\hat{n}_c(t+\Delta t)$. Following [6], we compute a rotation vector $\vec{\Omega}_c = \boldsymbol{I}_c^{-1}.\boldsymbol{L}_c$ where $\boldsymbol{I}_c = \sum_{v\in c}\left[(\vec{r}_v - \vec{R}_c)^2\boldsymbol{I}_3 - (\vec{r}_v - \vec{R}_c)\otimes(\vec{r}_v - \vec{R}_c)\right]/M_c$

Development • Supplementary information

is the moment of inertia of the cell and $\boldsymbol{L}_c = \sum_{v\in c}(\vec{r}_v - \vec{R}_c) \times (\vec{u}_v - \vec{U}_c)/M_c$ is the angular momentum of the cell. Here, $\vec{u}_v$ denotes the velocity of the vertex v, defined as $\vec{u}_v = (\vec{r}_v(t+\Delta t) - \vec{r}_v(t))/\Delta t$ and $\vec{U}_c$ denotes the velocity of the cell c defined as $\vec{U}_c = \sum_{v\in c}\vec{u}_v/M_c$. Finally, the rotation vector is obtained by projecting $\vec{\Omega}_c$ to $\hat{n}_c(t+\Delta t)$, which determines the second rotation matrix $\boldsymbol{R}_2 = \boldsymbol{\mathcal{R}}(\vec{\Omega}_c[\vec{\Omega}_c \cdot \hat{n}_c(t+\Delta t)]/|\vec{\Omega}_c|)$, where $\boldsymbol{\mathcal{R}}$ is the operator converting a rotation vector to the corresponding rotation matrix. To complete the second step we rotate the nematic by $\boldsymbol{R}_2$. The local reference frame of each cell is also corotated in the same manner.

II. MODEL PARAMETERS

Our model of Hydra tissue contains a number of parameters that we have to choose appropriately. Below, we explain the rationale behind this and Table 1 summarizes all the parameter values used. The mechanical parameters K, Γ, and Λ were first constrained such that the cellular network is in the solid phase [7, 8], since the behavior of the Hydra during early regeneration appears to be elastic. Furthermore, following our observation that cell rearrangements are rare even during the large stretching events, as well as motivated by recent experimental observations in other epithelial tissues [9, 10], we decided to set the parameters such that the two-dimensional Poisson ratio of the cellular network is negative. To this end we follow the analysis of the vertex model in [11] and set $\overline{\Lambda} = \Lambda/(KA_0^{3/2}) = 0$ and $\overline{\Gamma} = \Gamma/(KA_0) = 0.1$. We use $\sqrt{A_0}$ as length unit, and KA_0^2 as a unit of energy. Furthermore, we set the time unit in simulations so that the elastic cell relaxation time-scale is $\overline{\tau} = KA_0/\gamma = 1$. Although we do not have a precise measurement in Hydra, we expect that this time-scale corresponds to a value somewhere in the range of 1s - 1min in experiments. For the choice of bending modulus value $\beta = 0.1KA_0^{3/2}$ we did not have a constraint from literature or experiments so we chose an intermediate value that was sufficient to prevent strong bending at scale of single cells, but not too large as to dominate the tissue mechanics.

We chose the cell active stress amplitude to be $\zeta_M = -0.5\ K\ A_0^{3/2}$, such that the isotropic strain profile we obtain in the simulation of a single stretching event is comparable to the one experimentally observed in Hydra (Fig. S4).

The parameters controlling degradation and diffusion of morphogen define a length-scale over which the morphogen diffuses from the source $\lambda = \sqrt{D/(A_0 r_-)}$. We take a value of $r_- = 0.0007/\overline{\tau}$. Motivated by observations of the decay time-scale of the 'head forming potential' in regenerating Hydra reported in Ref. [12] we take the corresponding time-scale $\tau_\phi = 1/r_-$ to be 10hr. This also sets the elastic cell relaxation time-scale, introduced above, to be 25s. We next choose the morphogen diffusion coefficient to be $D = 0.0063\sqrt{A_0}/\overline{\tau}$, such that $\lambda \approx 3\sqrt{A_0}$. Furthermore, the total amount of morphogen in the system N_0 is proportional to r_+/r_-. We now choose to measure molecule number in units where $r_+ = 1/\overline{\tau}$ and r_- is chosen as above. Therefore, in our simulations we set $r_+ = 1$.

In order to characterize how the model parameters influence the competition between the nematic neighbor alignment and alignment to morphogen gradients, we construct a dimensionless number that quantifies this competition. Assuming that the region in which morphogen is produced is small compared to the region over which it diffuses from the source, we can estimate the morphogen concentration amplitude as the ratio between the total amount of morphogen and the area over which the morphogen diffuses A_d, which is proportional to λ^2. Therefore, we expect the amplitude of the morphogen concentration to scale as $N_0/A_d = r_+/\left(r_-\lambda^2\right)$, and at distance x from the morphogen source, the morphogen alignment term will scale as $\alpha\left((N_0/A_d)/\lambda\right)^2 e^{-2x/\lambda} \sim \alpha\left(r_+/\left(r_-\lambda^3\right)\right)^2 e^{-2x/\lambda} \sim \alpha r_+^2 r_-/\left(D^3 A_0^{3/2}\right) e^{-2x/\lambda}$. Now, we define a dimensionless function $S(x)$ as a ratio of morphogen alignment strength to the neighbor alignment rate $1/\tau_q$ at distance x from the source:

$$S(x) = \frac{\tau_q \alpha r_- r_+^2}{D^3 A_0^{3/2}} e^{-2x/\lambda}. \tag{14}$$

In the region where $S(x) \ll 1$, nematic neighbor alignment dominates, while in the region where $S(x) \gg 1$ nematic dynamics is dominated by the alignment to the morphogen gradient. The length-scale Λ that separates these two regimes can be identified by setting $S(\Lambda) = 1$, so that

$$\Lambda = \frac{\lambda}{2} \ln\left(\frac{\tau_q \alpha r_+^2 r_-}{D^3 A_0^{3/2}}\right). \tag{15}$$

Now, we can constrain the choice of remaining parameters τ_q and α by setting $\Lambda \approx 6.8\sqrt{A_0}$, which is comparable to λ so that alignment to the morphogen gradient is dominant within few cells from the source, while neighbor alignment is dominant further away from the source. Finally, we choose τ_q to correspond to 13min in experiments, which determines uniquely the value for α. Note that our choice of τ_q is such that $1/\tau_q$ is longer than duration of stretching events.

Finally, we choose to simulate a single stretching event by turning on the active stress magnitude uniformly in all cells with,

$$\zeta(t) = \zeta_M \exp\left(-\left(\frac{t - t_M}{2\sigma_M}\right)^2\right) \tag{16}$$

The duration of the pulse ΔT_ζ, defined as the full width at half maximum $\Delta T_\zeta = 2.355\sigma_M$ is chosen to correspond to about 8min. When simulating multiple stretching events spacing between subsequent pulses i and $i+1$: $T_\zeta = t_{M,i+1} - t_{M,i}$ is taken to correspond to 1hr.

Development • Supplementary information

Model parameter	Expression	Value	Value choice rationale
Cell length-scale	$\sqrt{A_0}$	1	unit length
Cell energy-scale	KA_0^2	1	unit energy
Elastic relaxation time-scale	$\overline{\tau} = KA_0/\gamma$	1	unit time
Normalized bond tension	$\overline{\Lambda} = \Lambda/(KA_0^{3/2})$	0	negative vertex model Poisson ratio
Normalized perimeter elasticity	$\overline{\Gamma} = \Gamma/(KA_0)$	0.1	negative vertex model Poisson ratio
Normalized bending modulus	$\overline{\beta} = \beta/(KA_0^{3/2})$	0.1	
Normalized active stress magnitude	$\overline{\zeta}_M = \zeta_M/(KA_0^{3/2})$	−0.5	reproduces experimentally observed strain
Time interval between activity pulses	$T_\zeta/\overline{\tau}$	160	corresponds to about 1 hr
Duration of activity pulse (FWHM)	$\Delta T_\zeta/\overline{\tau}$	18.84	corresponds to about 8min
Morphogen degradation rate	$r_-\overline{\tau}$	0.0007	corresponds to 1/(10hr)
Morphogen production rate	$r_+\overline{\tau}$	1	corresponds to about 1/(25s)
Morphogen diffusion coefficient	$D\overline{\tau}/\sqrt{A_0}$	0.0063	diffusion length scale of $\lambda = 3\sqrt{A_0}$
Nematic alignment rate	$\overline{\tau}/\tau_q$	0.03	corresponds to 13min
Nematic-morphogen coupling strength	$\alpha\overline{\tau}/A_0^3$	0.001	
Strain threshold for morphogen production	$\epsilon_{\rm th}$	0.25	

TABLE 1. Values of the model parameters used in the simulations. We summarize the rationale behind the choice in the last column. Correspondences to real times are based on experimental observations. For the parameters β, τ_q and α we did not have a strong constraint from literature or experiments and the value choice was based on the simulation outcome. The strain threshold value was systematically varied (Fig. S6). The value reported in the table corresponds to the value in Fig. 7C.

We sumarize the chosen values of the model parameters in Table 1.

III. SIMULATIONS OF REGENERATING HYDRA

A. Preparation of initial state

In order to perform simulations of stretching events in regenerating Hydra, as well as the dynamics of the nematic organisation and morphogen distribution we first have to prepare a state of the tissue that will serve as an initial condition from which we evolve the dynamical equations outlined above. We prepare the initial cellular network configuration for all simulations in this manuscipt by first generating a spherical tiling. To this end we start with an icosahedron and we apply alternating 4 "truncate" and 3 "dual" operations, as defined by the Conway polyhedron notation [13]. After the last "truncate" operation we have a cellular network made of 812 cells. The number of cells is chosen to be similar to the value in experiments. We then randomize the network by introducing stochastic fluctuations of the bond tension parameter Λ_b independently in each bond using

$$\frac{d\Lambda_b}{dt} = -k_\Lambda(\Lambda_b - \Lambda_0) + \sqrt{2k_\Lambda}\xi(t), \qquad (17)$$

where the first term corresponds to relaxation of Λ_b to a target value Λ_0 at a rate k_Λ, and the second term corresponds to fluctuations in Λ_b with $\xi(t)$ denoting gaussian white noise: $\langle\xi(t)\xi(t')\rangle = 2D_\xi\delta(t-t')$. We choose $k_\Lambda = 1, \Lambda_0 = -0.15, D_\xi = 0.35$. We proceed to randomize the cellular network for $T_{\rm rand.} = 100\overline{\tau}$. During the randomization process cells that become very small in area are extruded from the network and by the end of randomisation we have 804 cells remaining. Finally, we revert the bond tension value in all bonds to the uniform value we use in the actual simulations (Table 1), and minimize the vertex model energy to the nearest local minimum.

B. Strain focusing at nematic defects

We simulate a single stretching event during Hydra regeneration to characterize the strain pattern induced by global contraction of the muscle fibers whose orientation is described by the nematic field. Since stretching events typically last only several minutes (see main text and Fig. 3D), which is faster than the time-scale on which the nematic fiber pattern evolves [14], we do not consider here changes in the nematic pattern during a single event. We thus keep the nematic in each cell fixed, except for changes associated with convection and co-rotation of the cells as the tissue deforms.

We consider two nematic configurations. In the first case, we emulate the initial fiber pattern observed just after the tissue fragment folds into a spheroid, where a large part of the tissue surface is covered by an ordered nematic array without defects, which surrounds a disordered region of total nematic charge +2 (Fig. 1 B). In the disordered region, we take nematic in each cell to be oriented randomly in the cell tangent plane. Different realisations of disorder correspond to different sampling of the nematic orientations in the disordered region. In

Development • Supplementary information

the second case, we consider a fully ordered nematic field with one +1 defect and a pair of +1/2 defects on the opposite side, emulating the nematic fiber pattern observed in regenerating Hydra around $10-20$ hours after excision (Fig 1 C). In both cases, we activate the contraction in all cells simultaneously, following Eq. 16.

C. Mechanochemical model of Hydra regeneration

We simulate the dynamics of the proposed feedback mechanims that involves strain focusing during stretching events, mechanosensitive morphogen production and nematic alignment with morphogen gradients (Fig. 7A). The simulations are intitiated from the configuration of cells desribed above, with a large disordered region in the nematic field with a total charge of +2, emulating the early organization in the folded Hydra spheroid.

In order to explore the space of regeneration outcomes we vary the morphogen production threshold ϵ_{th} and the coupling strength between the nematic field and the morphogen gradients α. Changing of α in the range between $\alpha = 10^{-4}$ and $\alpha = 5 \cdot 10^{-2}$ corresponds to varying the length-scale Λ in the range between $\Lambda \approx 3\sqrt{A_0}$ and $\Lambda \approx 13\sqrt{A_0}$. Here, for our choice of the lower limit, Λ corresponds to several cells, whereas for the upper limit, Λ covers a bit more than a quarter of the simulation sphere circumference. We vary the morphogen production threshold over the range $\epsilon_{th} = 0.2 - 0.35$. We generate an ensemble of 100 initial states using the initial cellular packing (obtained as described in IV.A) with a different random configuration of the nematic field orientation in the disordered region in each initial state. We performed a total of 4200 simulations for different values of α and ϵ_{th}, out of which 7 failed, and we report the data from the successful simulations (Fig. S7). In addition, we also consider the special cases with either no morphogen alignment ($\alpha = 0$) or no morphogen production ($\epsilon_{th} \to \infty$) in the parameters we consider. For these special cases we generated an ensemble of 10 initial states. All 140 simulations of these special cases were successful.

We scan this range of model parameters and record the state of the system for the ensemble of initial states after 30 contractions (Fig. S7), corresponding to the typical regeneration time in experiments ($\approx 30hr$). We find that simulations with no morphogen production or no coupling of the nematic to morphogen gradients lead to solutions containing only +1/2 defects, whereas decreasing the threshold strain for morphogen production and increasing the morphogen coupling strength, yield stabilized +1 defects (Fig. 7C, S7). The appearance of solutions completely devoid of +1 defects at high values of the production threshold ϵ_{th}, is easy to understand as reduced morphogen production in the system makes it harder to stabilize +1 defects. Furthermore, for each value of ϵ_{th}, increasing α leads to an increasing number of +1 defects, likely due to easier nucleation and stabilisation of +1 defects in the presence of stronger nematic coupling to the morphogen gradient. The frequency of samples having excess defects, including negative -1/2 defects, however, changes non-monotonically. At low values of α we sometimes find long-lasting (i.e. longer than 30 contractions) -1/2 defects, since the resolution of excess defects through nearest neighbor coupling is slow at long distances. At intermediate values of α, these -1/2 defects do not form since coupling to the morphogen diffusing radially from +1 defect regions enhances the nematic ordering. Finally, at high values of α, additional +1/2 and -1/2 defects form, unlike in experiments. This happens because for large value of Λ, the nematic field can be influenced by the overlapping profiles of morphogen diffusing from two production regions. The parameter choice we use in the main text falls in the intermediate regime, where coupling to the morphogen is sufficient to induce +1 defect(s) and enhance nematic ordering, but not too high as to frequently induce excess defects in the nematic field organization.

We note that in the intermediate parameter range, simulations initiated from different initial conditions can develop a +1 defect or a pair of +1/2 defects at a given actin focus, for the same values of the model parameters. In this parameter range we often obtain simulation results with an asymmetric defect configuration containing a single +1 defect at one focus and a pair of +1/2 defects at the opposite focus (Fig. S7). The statistics of outcome defect configurations in these simulations are indistinguishable from the outcome statistics that would be expected if the two foci were independent of each other. This observation suggests that symmetry breaking in the model is not caused by interaction between defects, but rather arises due to subtle differences in the initial cell packing and nematic pattern in the disordered regions near the two focal points.

[1] Denis L Weaire and Stefan Hutzler, *The physics of foams* (Oxford University Press, 1999).

[2] John R. Szymanski and Rafael Yuste, "Mapping the whole-body muscle activity of hydra vulgaris," Current Biology **29**, 1807–1817.e3 (2019).

[3] Jordi Comelles, Soumya Ss, Linjie Lu, Emilie Le Maout, S Anvitha, Guillaume Salbreux, Frank J¨ ulicher, Mandar M Inamdar, and Daniel Riveline, "Epithelial colonies in vitro elongate through collective effects," eLife **10**, e57730 (2021).

[4] Bert Hobmayer, Fabian Rentzsch, Kerstin Kuhn, Christoph M. Happel, Christoph Cramer Von Laue, Petra Snyder, Ute Rothbächer, and Thomas W. Holstein, "Wnt signalling molecules act in axis formation in

the diploblastic metazoan hydra," Nature **407**, 186–189 (2000).

[5] Pierre-Gilles De Gennes and Jacques Prost, *The physics of liquid crystals*, 83 (Oxford university press, 1993).

[6] Aboutaleb Amiri, Charlie Duclut, Frank Jülicher, and Marko Popović, "Random traction yielding transition in epithelial tissues," Physical Review Letters **131**, 188401 (2023).

[7] D. B. Staple, R. Farhadifar, J. C. Röper, B. Aigouy, S. Eaton, and F. Jülicher, "Mechanics and remodelling of cell packings in epithelia," The European Physical Journal E **33**, 117–127 (2010).

[8] Dapeng Bi, J. H. Lopez, J. M. Schwarz, and M. Lisa Manning, "A density-independent rigidity transition in biological tissues," Nature Physics **11**, 1074–1079 (2015).

[9] Natalie A Dye, Marko Popović, K Venkatesan Iyer, Jana F Fuhrmann, Romina Piscitello-Gómez, Suzanne Eaton, and Frank Jülicher, "Self-organized patterning of cell morphology via mechanosensitive feedback," eLife **10**, e57964 (2021).

[10] Romina Piscitello-Gómez, Franz S Gruber, Abhijeet Krishna, Charlie Duclut, Carl D Modes, Marko Popović, Frank Jülicher, Natalie A Dye, and Suzanne Eaton, "Core pcp mutations affect short-time mechanical properties but not tissue morphogenesis in the drosophila pupal wing," eLife **12**, e85581 (2023).

[11] Michael F. Staddon, Arthur Hernandez, Mark J. Bowick, Michael Moshe, and M. Cristina Marchetti, "The role of non-affine deformations in the elastic behavior of the cellular vertex model," Soft Matter **19**, 3080–3091 (2023).

[12] Harry K MacWilliams, "Hydra transplantation phenomena and the mechanism of hydra head regeneration: Ii. properties of the head activation," Developmental biology **96**, 239–257 (1983).

[13] "Conway notation for polyhedra," `https://www.georgehart.com/virtual-polyhedra/conway_notation.html`, [Accessed 14-05-2024].

[14] Yonit Maroudas-Sacks, Liora Garion, Lital Shani-Zerbib, Anton Livshits, Erez Braun, and Kinneret Keren, "Topological defects in the nematic order of actin fibres as organization centres of hydra morphogenesis," Nature Physics **17**, 251–259 (2021).

Development • Supplementary information

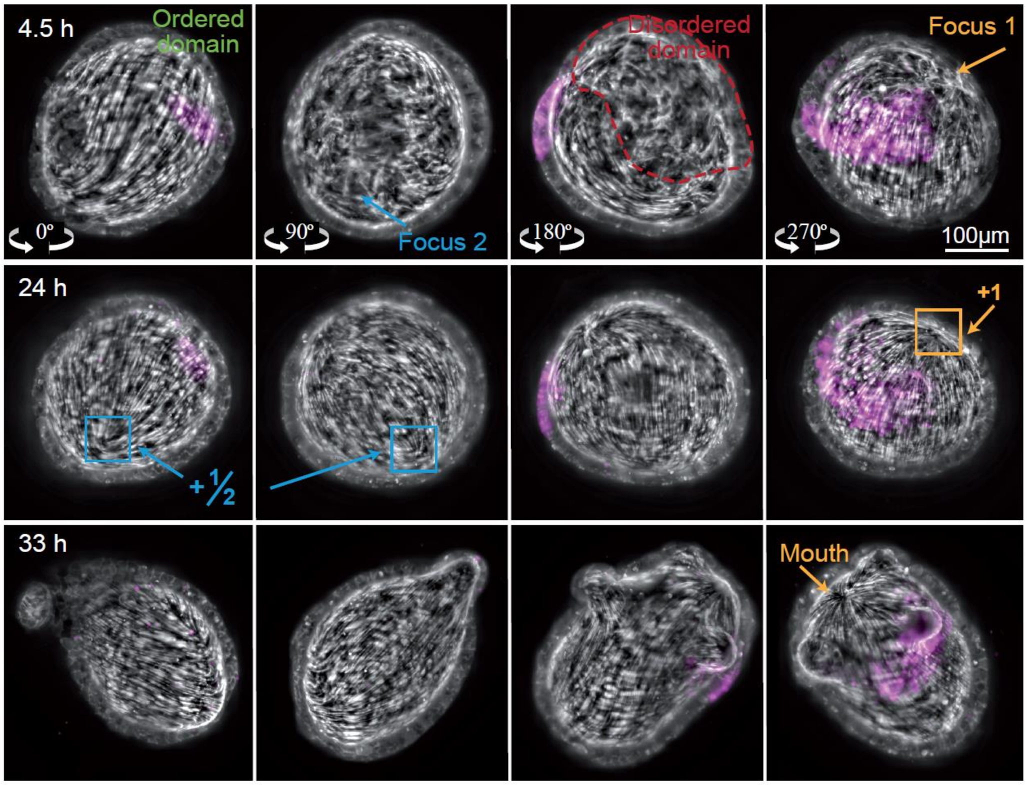

Fig. S1. 3D view of the actin fiber reorganization in a regenerating Hydra. Images from a time-lapse light sheet movie of a regenerating Hydra expressing Lifeact-GFP in the ectoderm, showing projected views from four different directions at early (top), intermediate (middle) and late (bottom) stages of the regeneration process. The two actin fiber foci are visible in the folded spheroid (top panels), and develop, respectively, into an aster-shaped +1 defect at the future head site (orange labels), and a pair of +½ defects at the future foot site (cyan labels). The actin fiber organization (gray) is shown with an overlay the photoactivated dye (magenta; Abberior CAGE 552), identifying the same group of cells over time. All images are maximum projection images of the light sheet stack acquired from the direction indicated, showing the fiber organization at the basal surface of the ectoderm.

Development • Supplementary information

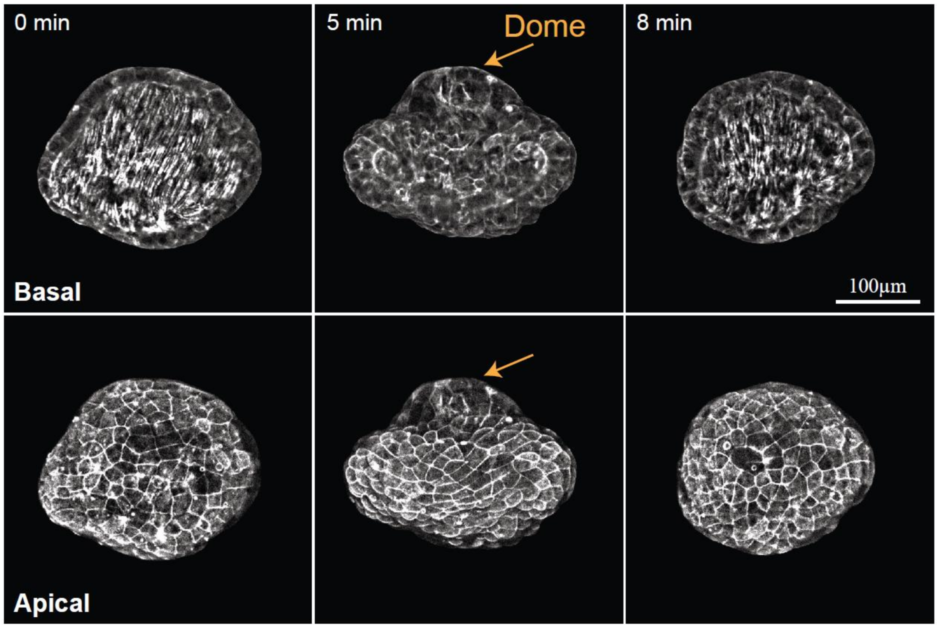

Fig. S2. Doming of the tissue during Hydra regeneration. Images from a time-lapse spinning-disk movie of a regenerating Hydra expressing Lifeact-GFP in the ectoderm, exhibiting doming of the tissue at a defect site during a large tissue stretching event. The images show the fiber organization at the basal surface (upper panels) and the cellular organization at the apical surface (lower panels) before (left), during (middle) and after (right) the event. The images are projected views of the ectodermal basal or apical surface, computationally generated from 3D spinning-disk confocal stacks. During the event, the tissue in the defect region undergoes localized stretching and exhibits an abrupt change in surface curvature resulting in the formation of a tissue dome (middle panels). Doming is suggestive of inhomogeneous or non-linear material properties that produce excessive stretching near the core of the event and induce out-of-plane deformations (Latorre et al., 2018).

Development • Supplementary information

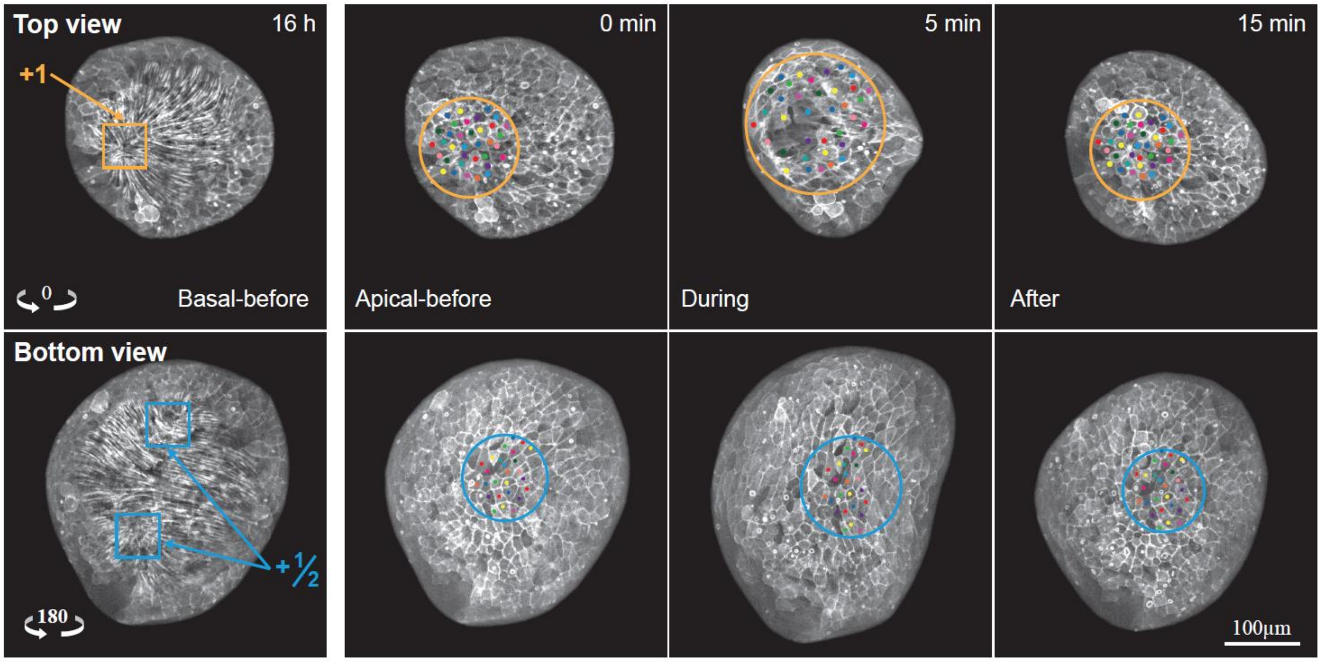

Fig. S3. Simultaneous mechanical strain focusing at both actin foci. Images from a time-lapse up-and-under spinning-disk movie of a regenerating Hydra, during a global deformation event exhibiting mechanical strain focusing at both actin foci. The top view shows the deformations at and around a +1 defect, whereas the bottom view displays the deformations at a pair of +½ defects. For each view, the left image depicts the actin fibers at the basal surface of the ectoderm, and the right images show the cellular organization at the apical surface before (left), during (middle) and after (right) the event, with colored dots marking manually-tracked individual cells. All images are projected views of the ectodermal basal or apical surface, computationally generated from 3D spinning-disk confocal stacks of regenerating tissues expressing Lifeact-GFP in the ectoderm.

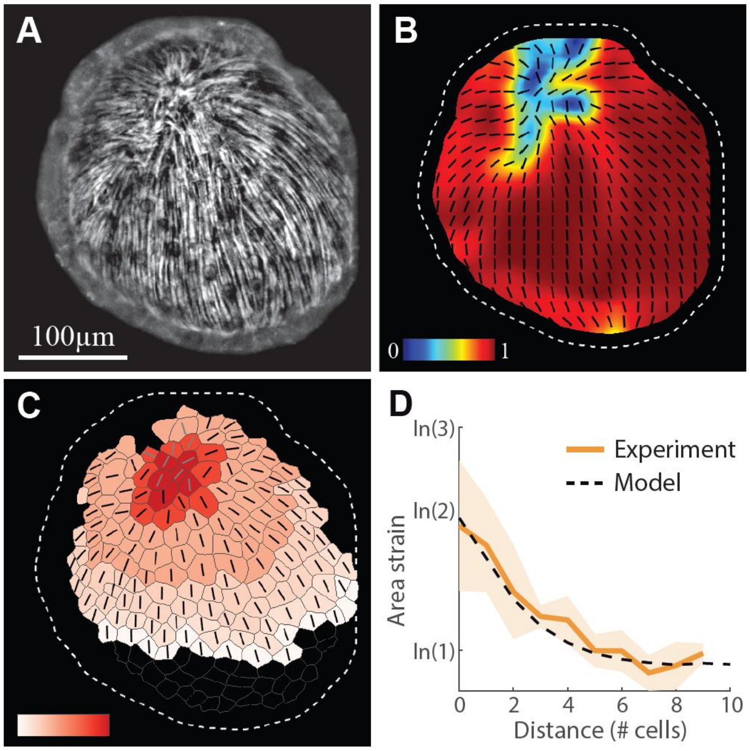

Fig. S4. The nematic fiber organization and cell area strain at an aster-shaped +1 defect. (A-C) Images from a time-lapse spinning-disk movie of a regenerating Hydra expressing Lifeact-GFP in the ectoderm (Fig. 3A, Movie 3), showing: (A) the actin fibers at the basal surface of the ectoderm (image reproduced from Fig. 3A), (B) the actin fiber nematic orientation (lines) and local nematic order parameter (color shading, see supplementary Materials and Methods [19]), and (C) a cell segmentation map with cells colored according to the logarithmic cell area strain at the peak of a stretching event (color shading, range: [-0.3, 0.78]), overlaid with the nematic fiber orientation (lines). The cell area strain is calculated by averaging over all cells at a given distance from the defect as described in (D). The tissue outline in (B,C) is indicated by a dashed line. (D) The logarithmic cell area strain at the peak of individual stretching events around a +1 defect site, as a function of distance from the focal point. The logarithmic strain is calculated as the log of the ratio of cell area at the peak of the event and before it, averaged for all cells at each distance. Distances are calculated as graph distances (degree of neighbor relation) between cells. The mean strain averaged over 8 events from Movie 3 (mean and std), is shown together with the model-simulated strain pattern (dashed line; Fig. 5C).

Development • Supplementary information

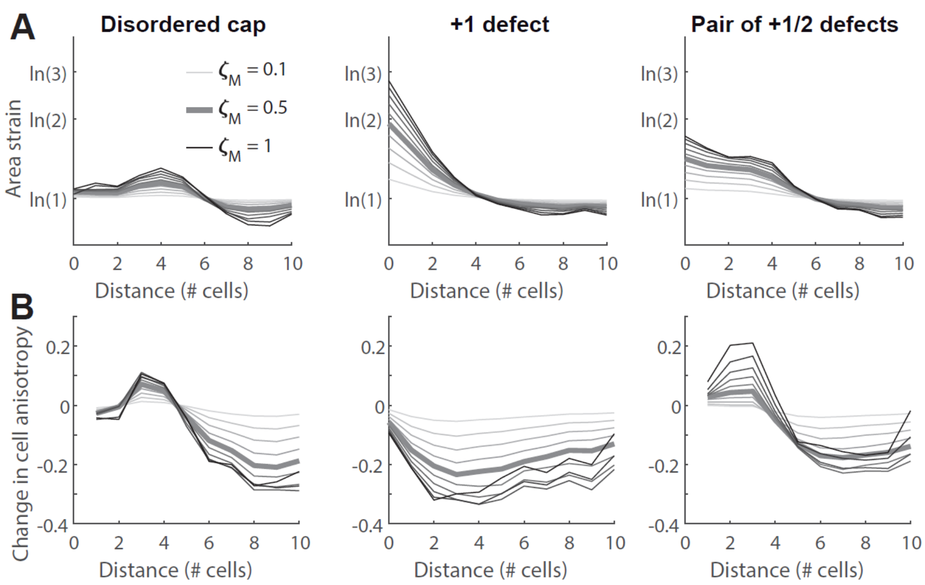

Fig. S5. Results of model simulations of mechanical strain focusing at actin foci for different values of the activation amplitude. Plots showing the average cell area strain (A) and change in cell anisotropy (B) during the peak of an activation event, as a function of distance in cells from the focus region in model simulations. The cell area strain is defined as $\epsilon_c = \ln\left(A_c/A_c^{ref}\right)$, where A_c is the cell area at the peak of the event and A_c^{ref} is the initial cell area. The change in cell anisotropy is defined as $\Delta Q_{rr} = Q_{rr} - Q_{rr}^{ref}$, where Q_{rr} and Q_{rr}^{ref} are the radial components (relative to the focus point) of the cell elongation tensor $\boldsymbol{Q}$ in the strained and relaxed states, respectively. Simulations of regenerating fragments at early stages of the regeneration process show cell stretching around the foci in the disordered cap regions (left panels). Simulations of regenerating fragments at later stages of the regeneration process show larger stretching around the +1 defect on one side (middle panels) and more moderate stretching at the pair of +½ defects at the opposite focus (right panels). The different lines depict the simulation results for different values of the activation amplitude $\bar{\zeta}_M$, going from 0.1 (light gray) to 1 (black) in steps of 0.1. Simulation results for $\bar{\zeta}_M = 0.5$, which is the value used in Figs. 5 and 7, are denoted by a thick line.

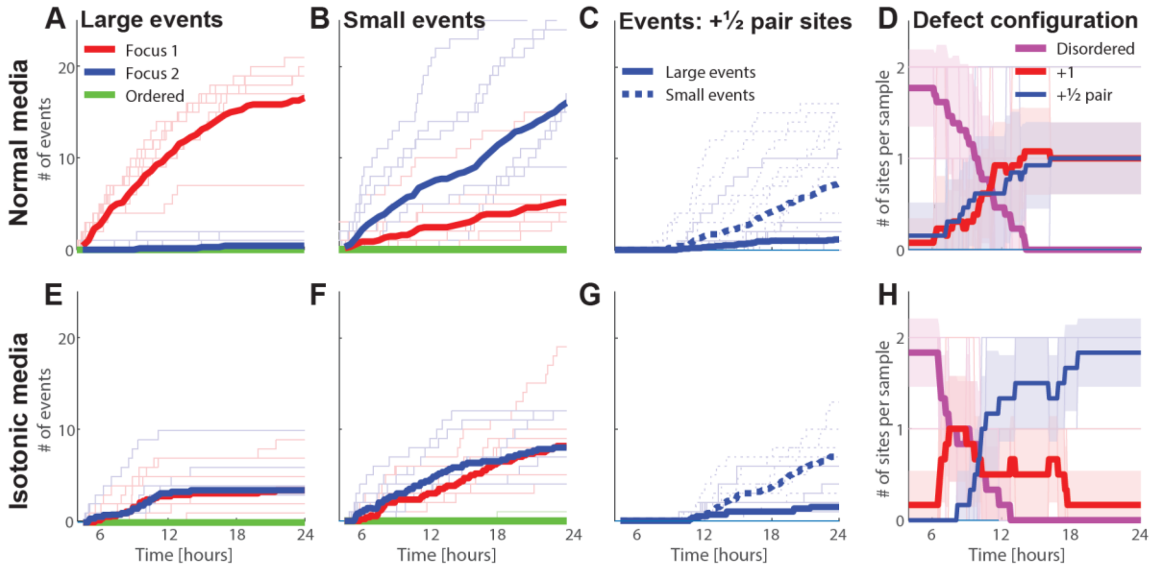

Fig. S6. Comparison of deformation events in normal media and in elevated medium osmolarity (+70mM sucrose). (A,B) Plots showing the cumulative number of large stretching and rupture events (A) or small stretching events (B), at different tissue locations in regenerating Hydra in normal media. (C) Plots showing the cumulative number of events at sites containing a pair of +½ defects in regenerating Hydra in normal media. (D) Plots showing the actin fiber organization in the foci as a function of time in regenerating Hydra in normal media. (E-H) Plots as in (A-D) for samples placed in elevated medium osmolarity (+70mM sucrose). In all plots, the thin lines depict results for individual movies, and the thick lines denote the mean over all samples. The shaded region in (D,H) denotes the standard deviation in the number of sites per sample with each type of fiber organization. Stretching and rupture events are manually characterized as in Figure 2. Large events are defined as events in which cells stretch more than ~2-fold in area or the tissue ruptures, whereas small events are defined as events with global cell strain changes with less than a ~2-fold increase in area. The data for normal media is from 7 high-resolution movies of regenerating Hydra in normal media, imaged from both sides using an up-and-under microscopy setup (data as in Figure 2). The data for elevated medium osmolarity is from 6 high-resolution up-and-under movies of regenerating fragments in Hydra medium supplemented with 70mM sucrose. Events are tracked during the first 24 hours of the regeneration process, starting ~4 hours after excision (after the spheroids fold and seal).

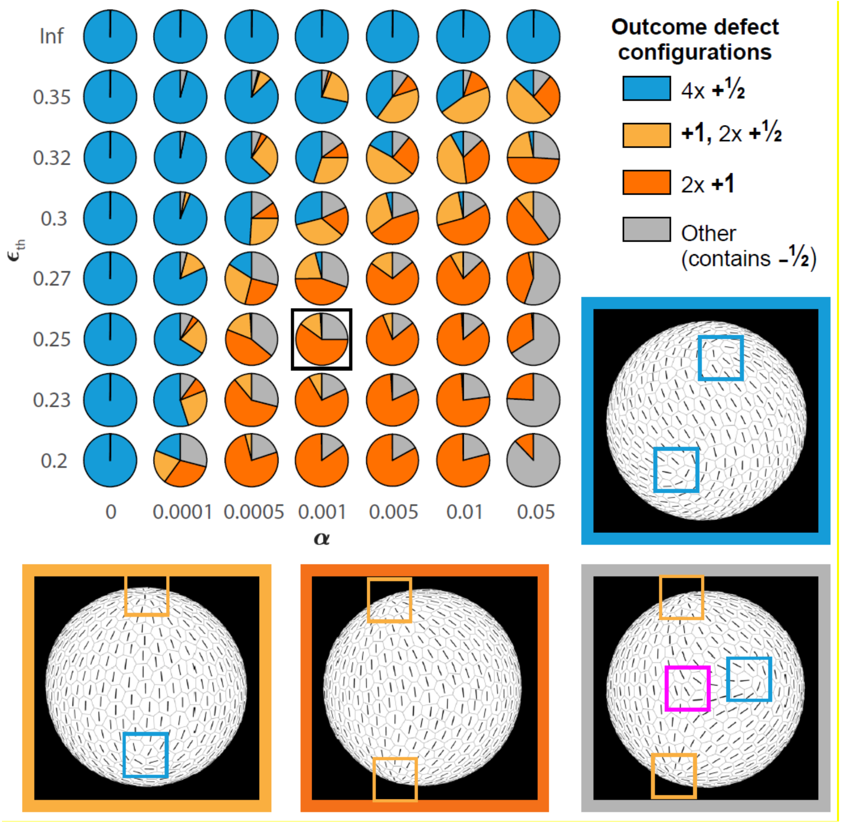

Fig. S7. Model predictions as a function of the morphogen production threshold ϵ_{th} and the nematic-morphogen coupling strength α. Regenerating Hydra tissues are simulated using the dynamical equations for the vertex positions, the nematic field and the morphogen concentration (see supplementary Materials and Methods), with different values of the morphogen production threshold ϵ_{th} and the nematic-morphogen coupling strength α, as indicated. The cell area strain threshold for morphogen production (ε_{th}) is varied from 0.2 to 0.35 or taken to be infinite (i.e. with no morphogen production), whereas the nematic-morphogen coupling strength (α) is varied from 0 (i.e. no coupling) to 0.05, while keeping all other model parameters fixed. The simulations are initiated with a large disordered domain of net charge +2 that is surrounded by a fully ordered region, emulating the actin fiber organization in regenerating fragments at early stages of the regeneration process and no morphogen (as in Fig. 5B). The simulation results for each pair of parameter values were evaluated for 100 different realizations of the nematic orientations in the initial disordered region, except in special cases with α=0 or $\varepsilon_{th}=\infty$, where only 10 different realizations were used (see supplementary Materials and Methods). The results of successful simulations were characterized into one of 4 possible outcome configurations, shown schematically next to the diagram of the regeneration outcomes. Point defects in the outcome configurations are surrounded by a colored box (+1 defects in gold, +½ defects in cyan, and -½ defects in magenta). For each parameter set, the outcome defect configurations are summarized in a pie plot. The parameter set used in Fig. 7 is indicated by a box. In the absence of morphogen-nematic coupling (α=0) or at high strain threshold values ($\varepsilon_{th}=\infty$), the nematic self-organizes into a configuration with four +½ defects.

Development • Supplementary information

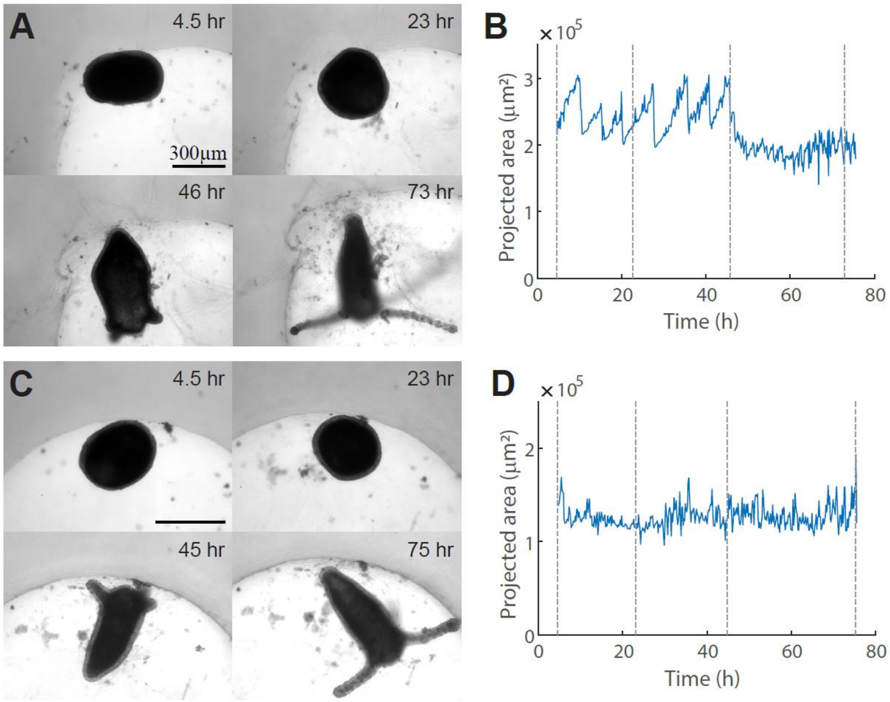

Fig. S8. Dynamics of regenerating Hydra tissue fragments. (A-D) Examples of the overall tissue dynamics of regenerating Hydra fragments in solution. (A,C) Images from bright-field movies of regenerating Hydra tissue fragments. (B,D) Graphs of the projected area of the regenerating tissues as a function of time for the movies shown in A and C, respectively. The dashed lines indicate the time of the images shown in A and C. While most samples exhibit a characteristic saw-tooth pattern (as in B), a fraction of samples (3 out of 26 examined) do not display this pattern, yet regenerate normally (as in C,D). Similarly, we have previously shown that tissue pieces regenerating on thin wires that pierce the tissue and generate a leaky spot, exhibit tissue dynamics with a few or no saw-tooth yet are still able to regenerate (Livshits et al., 2017).

Development • Supplementary information

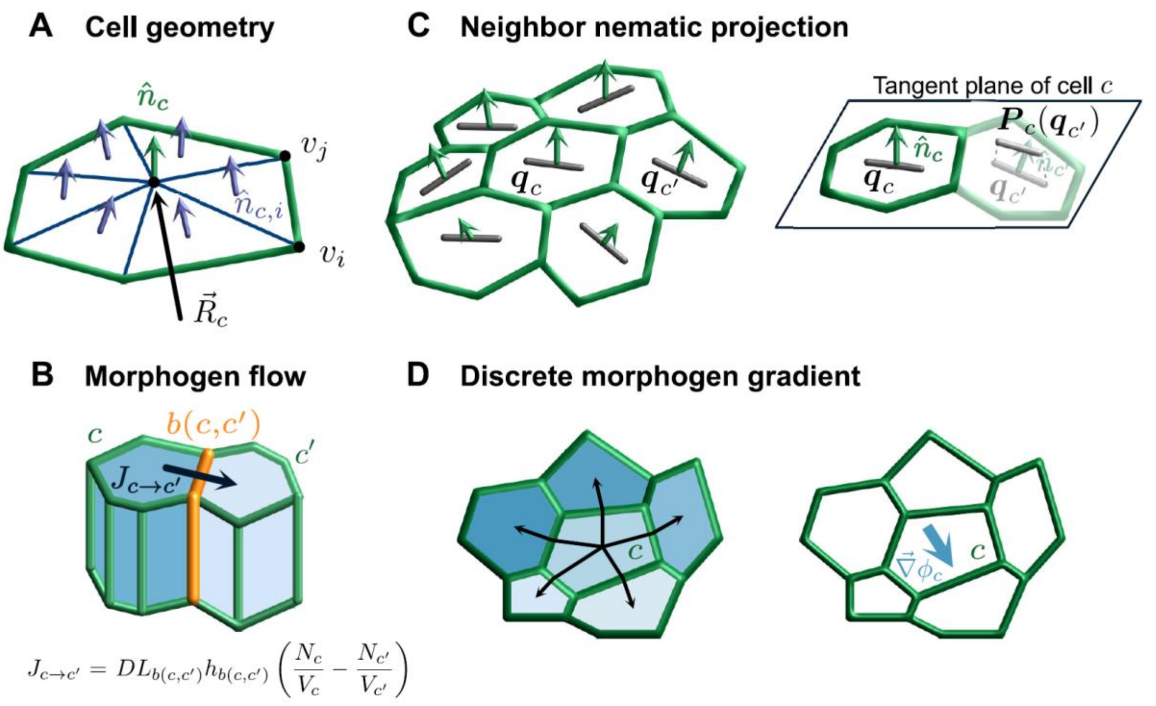

Fig. S9. Components of the mechanochemical model of Hydra regeneration. (A) Schematics of cell triangulation and the corresponding triangle normal vectors, whose average defines the cell normal vector. (B) Illustration of the three-dimensional picture of epithelial cells. Two neighboring cells share an interface of length $L_{b(c,c')}$ and height $h_{b(c,c')}$ through which morphogen can diffuse. The diffusion flux between neighboring cells is proportional to their morphogen concentration difference and the interface area. The transport coefficient D describes morphogen diffusion between the two cells. (C) Schematic of cell c and its neighbors with the orientation of the nematic in each cell indicated by black bars and the cell normal vectors indicated by green arrows. In order to compute $\langle \boldsymbol{q} \rangle_c$ we calculate for the nematic tensor in each of the neighboring cells $\boldsymbol{q}_{c'}$ its projection $\boldsymbol{P}_c(\boldsymbol{q}_{c'})$ in the tangent plane of cell c, and then average all the projected tensors in the tangent plane. (D) Illustration of the discrete morphogen gradient in cell c. The morphogen concentration ϕ values in each cell are indicated in blue color, with darker colors corresponding to higher values. The discrete morphogen gradient in cell c is defined as the vector $\vec{\nabla}\phi_c$ that minimizes the error in the relation $\phi_{c'} = \phi_c + \vec{\nabla}\phi_c \cdot \vec{r}_{c,c'}$ simultaneously for all neighboring cells c'.

Development • Supplementary information

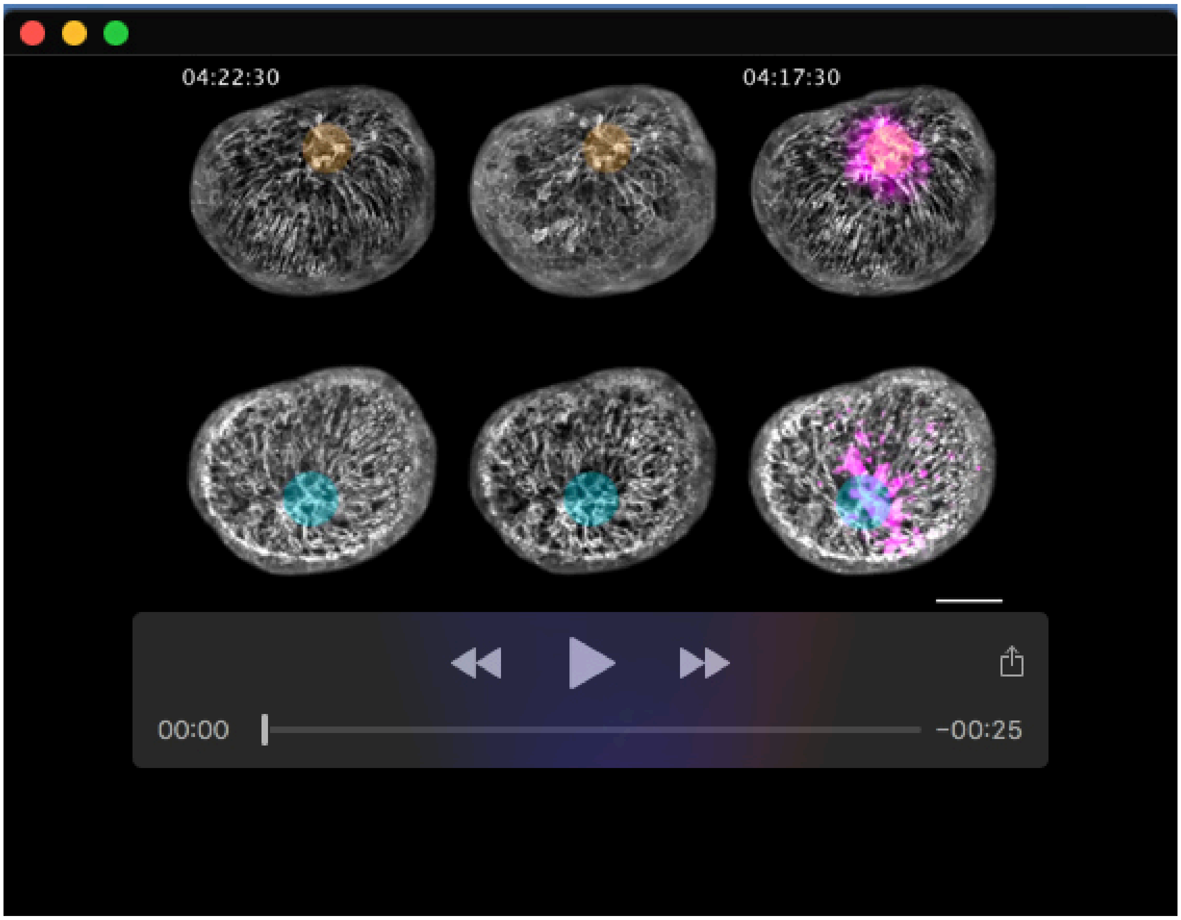

Movie 1. Foci of the actin fiber organization develop into topological defects localized at the regenerated head and foot regions

Time-lapse movies of a regenerating Hydra spheroid expressing Lifeact-GFP in the ectoderm acquired simultaneously from two opposite sides of the tissue using an up-and-under spinning-disk confocal microscope (Fig. 1C; upper panels: bottom 20x objective; lower panels: top 10x objective). The two regions around the foci of the actin fiber organization were labeled by laser-induced uncaging of Abberior CAGE 552 in the folded spheroid ~3 hours after excision, allowing us to follow the two focus regions throughout the regeneration process. An aster-shaped +1 defect emerges in the focus region at the future head site (orange labels), while a pair of +½ defects emerge in the second focus at the future foot site (cyan labels). The locations of the actin foci are determined manually, and depicted by dots that are replaced by square outlines once well-defined point defects appear. The images show computationally generated projected views of the ectodermal basal surface (left), the ectodermal apical surface (middle), and the basal layer with an overlay of the photoactivated dye (magenta; right). The images were centered to correct for movements of the whole tissue. The elapsed time from excision is displayed (hh:mm), and the scale bar is 100 µm.

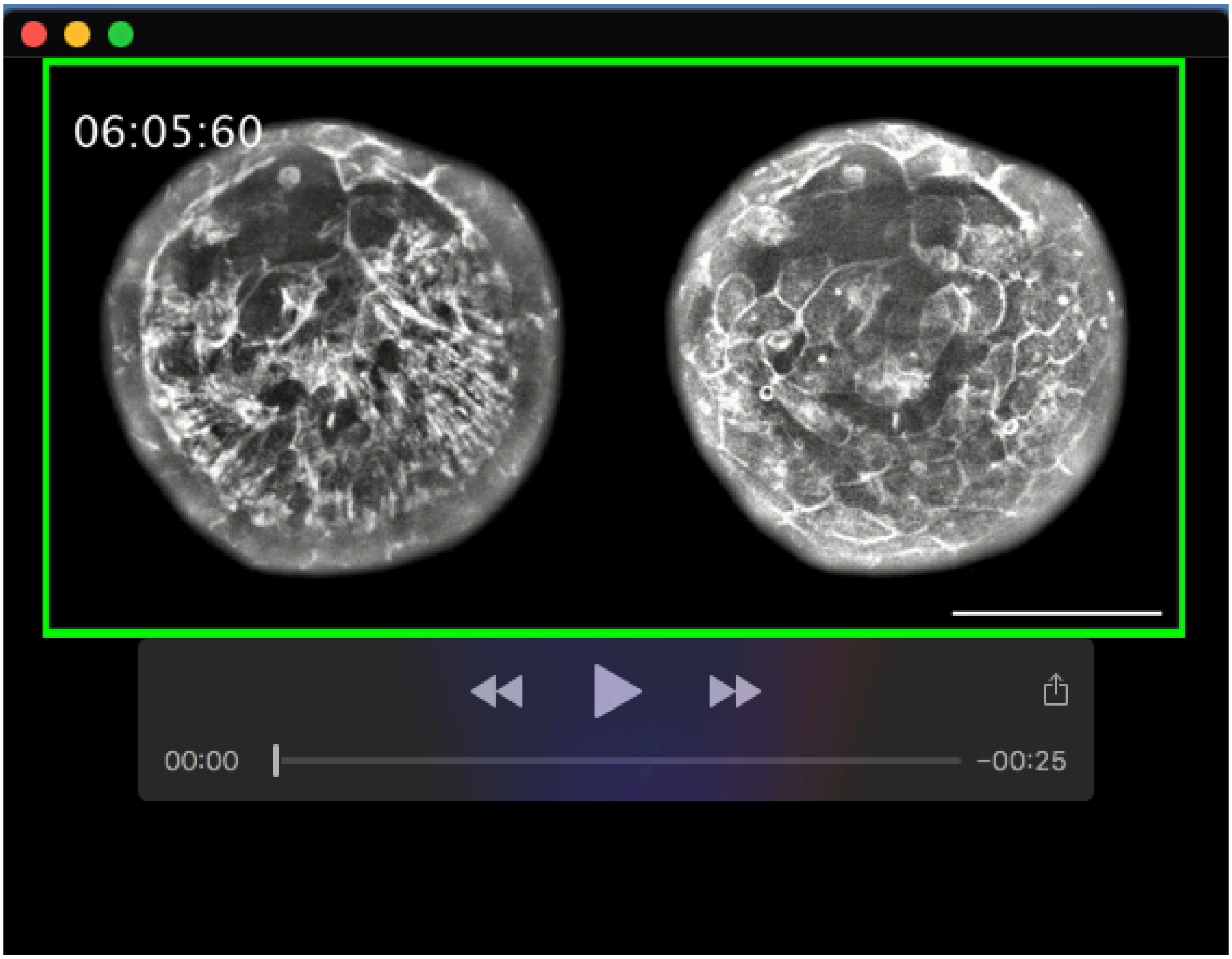

Movie 2. Mechanical strain focusing at the future head region.

High-resolution time-lapse, spinning-disk confocal movie acquired every 20 seconds of a regenerating tissue fragment expressing Lifeact-GFP in the ectoderm. The tissue spheroid is oriented with its future head region facing the objective. The images show computationally generated projected views of the fiber organization at the ectodermal basal surface (left) and the cellular organization at the ectodermal apical surface (right). Deformation events involving mechanical strain focusing at the future head site are indicated by green frames, and are slowed down to better demonstrate the tissue deformations. The elapsed time from excision is displayed (hh:mm:ss), and the scale bar is 100 µm.

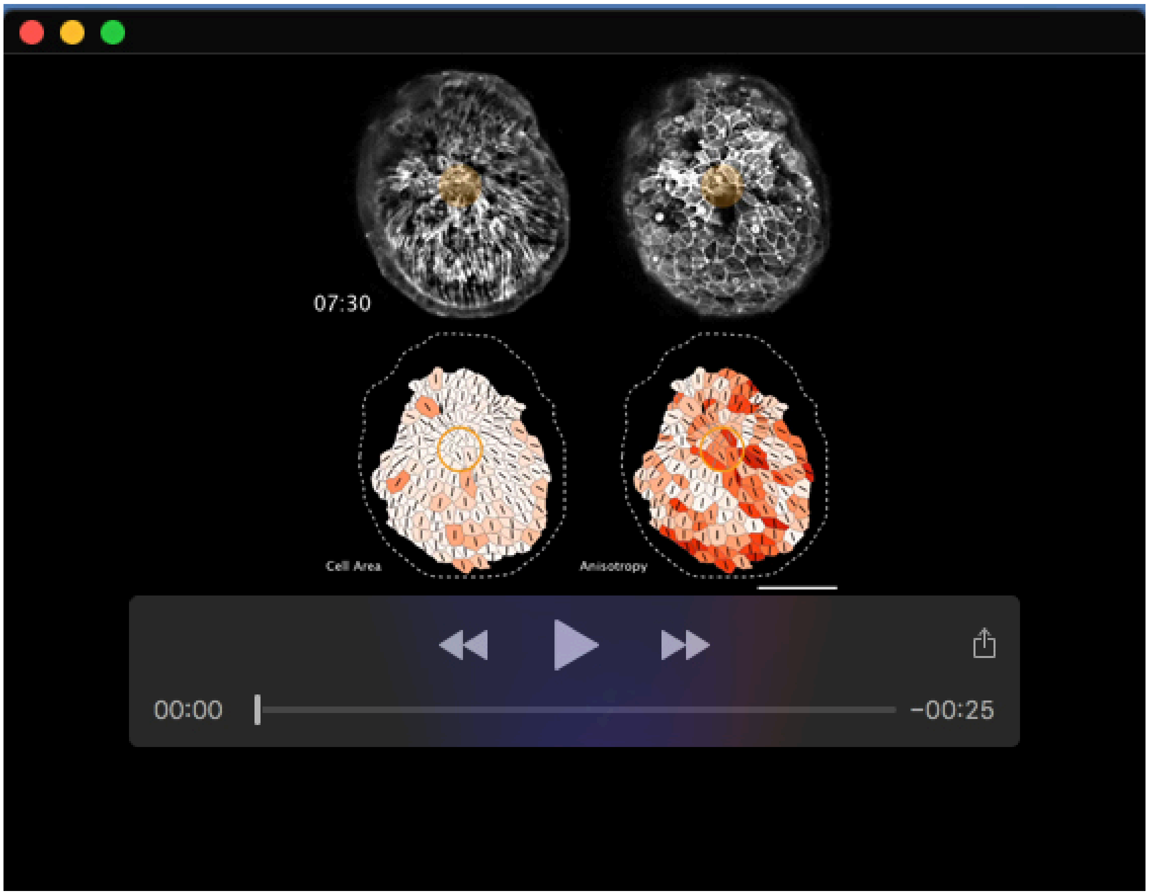

Movie 3. Characterization of mechanical strain around the future head site in a regenerating tissue spheroid.

Time-lapse, spinning-disk confocal movie of a regenerating tissue fragment expressing Lifeact-GFP in the ectoderm (Fig. 3A). The tissue spheroid was initially oriented with its future head region facing the objective, showing the focus region that develops into a +1 defect (orange labels). The images show computationally generated projected views of the fiber organization at the ectodermal basal surface (upper left), the cellular organization at the ectodermal apical surface (upper right), and cell segmentation maps depicting ectodermal cells colored according to their area (lower left; Range (white to red): 300-1200μm^2) or cell anisotropy (lower right; Range (white to red): Q=0.1-0.65). The nematic orientation of the fibers in each cell is overlaid on the segmentation maps. Stretching events leading to mechanical strain focusing at the future head side are indicated by green frames, and are slowed down to better demonstrate the tissue deformations. The elapsed time from excision is displayed (hh:mm), and the scale bar is 100 µm.

Development • Supplementary information

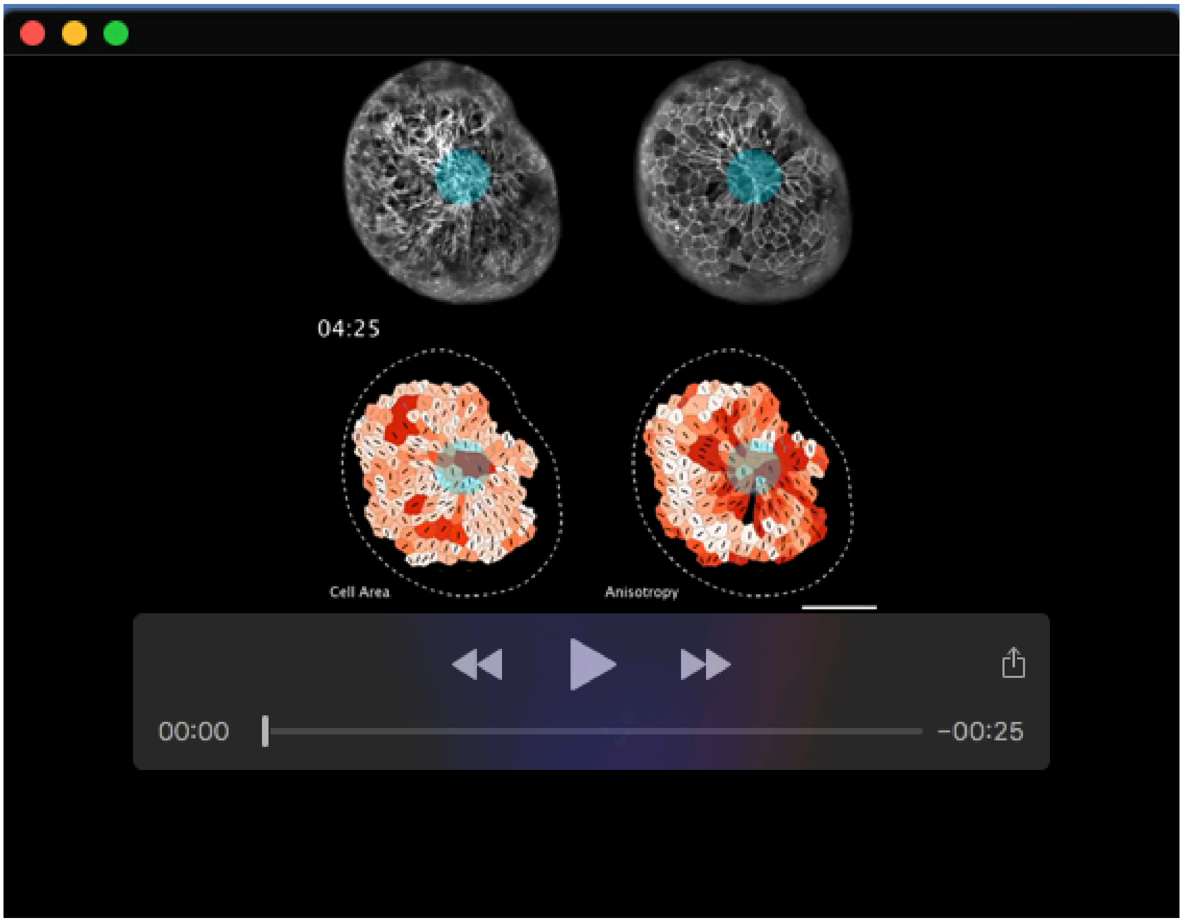

Movie 4. Characterization of mechanical strain around the future foot region in a regenerating tissue spheroid.

Time-lapse, spinning-disk confocal movie of a regenerating tissue fragment expressing Lifeact-GFP in the ectoderm (Fig. 3H). The tissue spheroid was oriented with its future foot region facing the objective, showing the focus region that develops into a pair of +½ defects at the future foot region (cyan labels). The images show computationally generated projected views of the fiber organization at the ectodermal basal surface (upper left), the cellular organization at the ectodermal apical surface (upper right), and cell segmentation maps depicting ectodermal cells colored according to their area (lower left; Range (white to red): 300-1200 μm^2) or cell anisotropy (lower right; Range (white to red): Q=0.1-0.65). The nematic orientation of the fibers in each cell is overlaid on the segmentation maps. The images were centered to correct for movements of the whole tissue. The elapsed time from excision is displayed (hh:mm), and the scale bar is 100 μm.

Development • Supplementary information

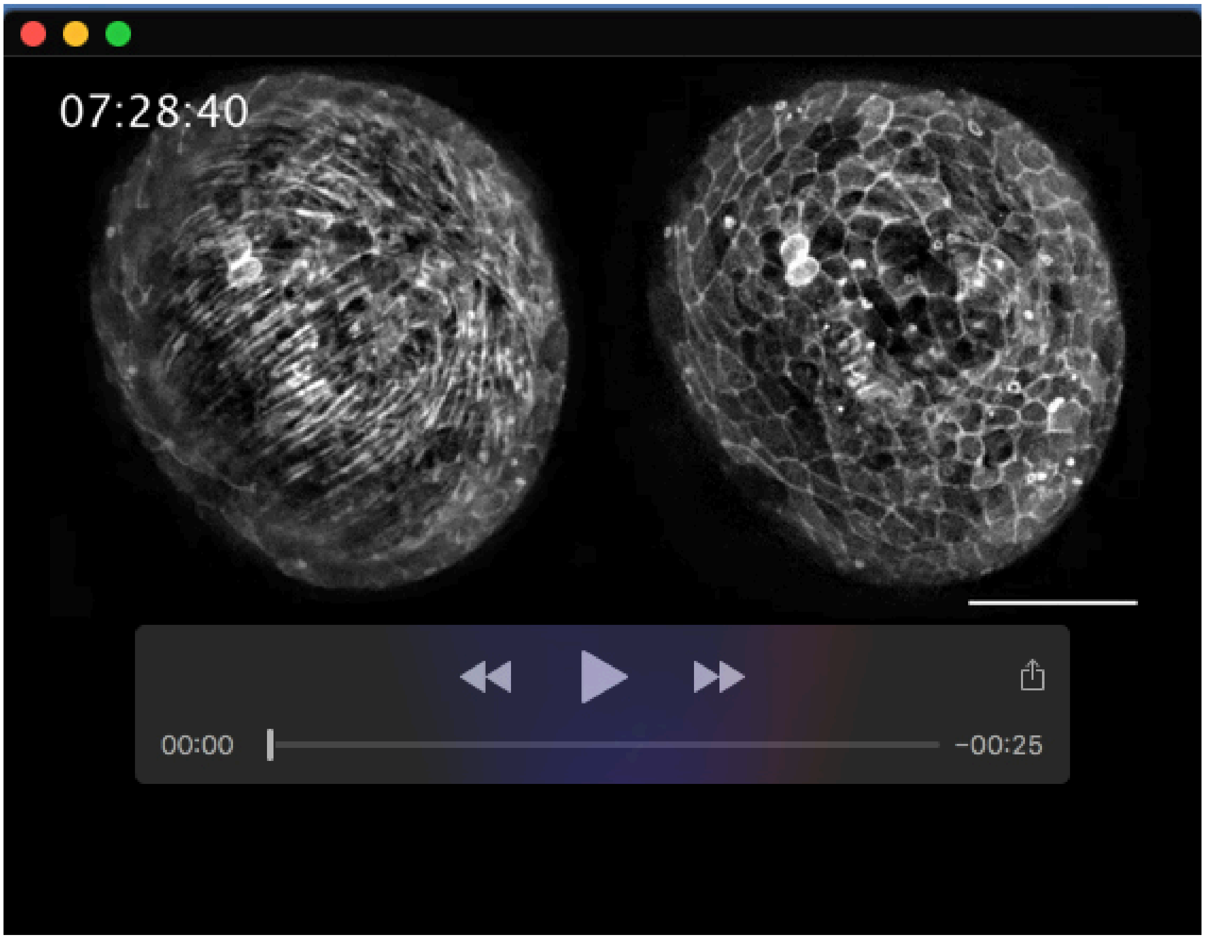

Movie 5. The formation of a rupture hole during a large stretching event at the focus of the actin fiber organization

High-resolution time-lapse, spinning-disk confocal movie acquired every 20 seconds showing the formation of a rupture hole in a regenerating Hydra expressing Lifeact-GFP in the ectoderm (Fig. 4B). The rupture occurs during a large stretching event, initiating as an elongated crack along ectodermal cell-cell junctions, rapidly becoming circular, and sealing back within minutes. The movie shows additional large stretching events (indicated by green frames), with mechanical strain focusing recurring at the same location, at the focus site of the ectodermal actin fibers. The events are slowed down to better demonstrate the tissue deformations. The images show computationally generated projected views of the fiber organization at the ectodermal basal surface (left) and the cellular organization at the ectodermal apical surface (right). The elapsed time from excision is displayed (hh:mm:ss), and the scale bar is 100 µm.

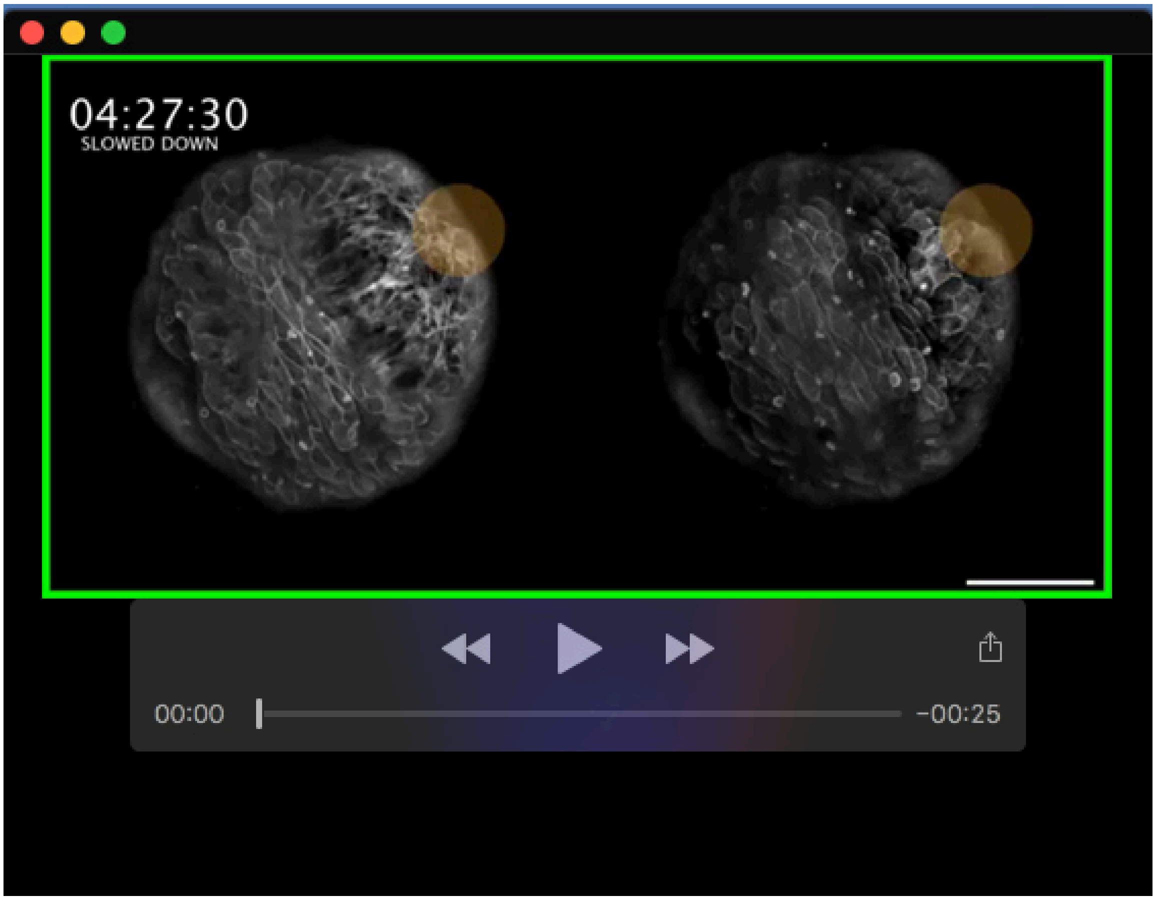

Movie 6. An extremely large rupture hole in a regenerating tissue spheroid

Time-lapse, spinning-disk confocal movie showing a regenerating tissue spheroid expressing Lifeact-GFP in the ectoderm that experiences two extremely large rupture holes lasting for an extended period of time (~1 hour; Fig. 4C). The movie shows that the tissue spheroid is nevertheless able to successfully regenerate into a mature Hydra. The rupture holes form at the +1 defect region at the future head site (orange labels). Large deformation events involving mechanical strain focusing and rupture hole formation recur at the same location and are indicated by green frames, and are slowed down to better demonstrate the tissue deformations. Later, large deformation events that are observed from the side (since the tissue rotated and the foci regions are not visible) are indicated by light green frames. The images show the computationally generated projected views of fiber organization at the ectodermal basal surface (left) and the cellular organization at the ectodermal apical surface (right). The images were centered to correct for movements of the whole tissue. The elapsed time from excision is displayed (hh:mm), and the scale bar is 100 µm.

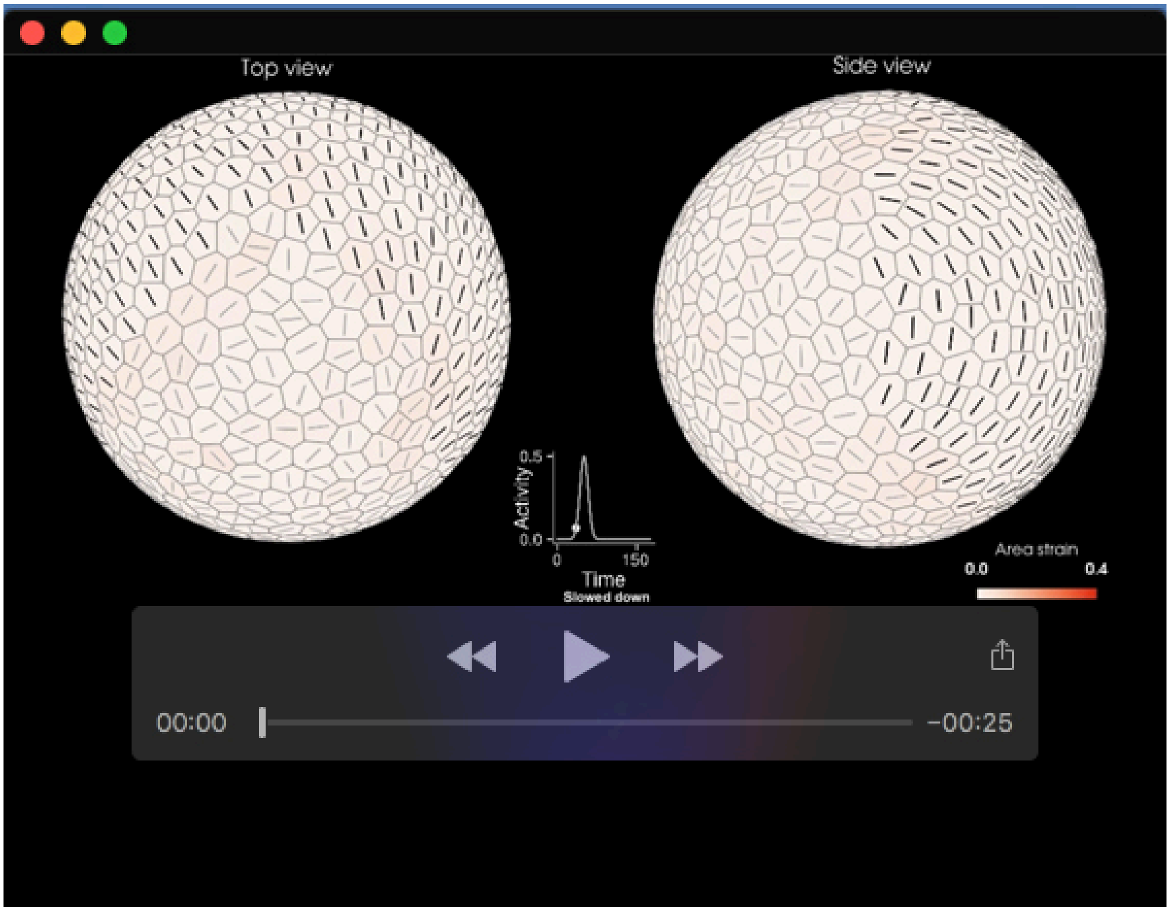

Movie 7. Simulation of Hydra tissue mechanics during global activation of fiber contraction in a spheroid with a large disordered domain.

The movie depicts the results of simulations of a transient activation of global fiber contraction in a Hydra tissue spheroid with a nematic configuration containing a large disordered domain of net charge +2 surrounded by a fully ordered region, emulating the nematic pattern at the early stages of regeneration (Fig. 5B). The tissue spheroid is modeled as a deformable shell that contains an embedded nematic field that experiences a time-dependent global activation of contraction (bottom inset). The images show cell segmentation maps depicting cells colored according to the cell area strain defined as $\epsilon_c = \ln\left(A_c/A_c^{ref}\right)$, where A_c is the cell area and A_c^{ref} is the initial cell area, overlaid with lines indicating the orientation of the embedded nematic field in each cell (black- ordered region; gray- disordered regions). The spheroid is shown from a top view centered on one of the actin foci and a side view. The active stress generated within each cell is aligned with the nematic field in that cell, and global activation leads to mechanical strain focusing around the two foci.

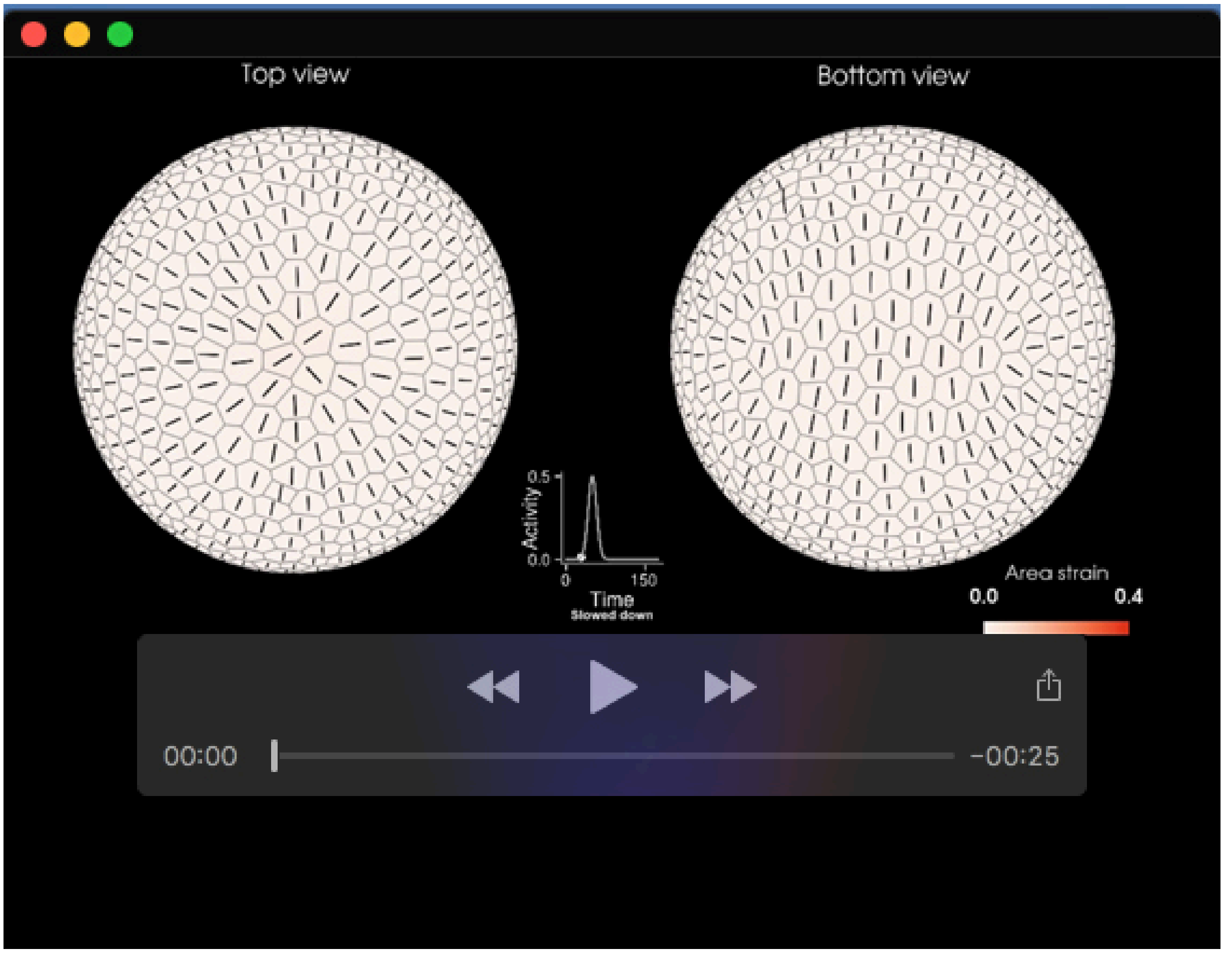

Movie 8. Simulation of Hydra tissue mechanics during global activation of fiber contraction in a spheroid with a +1 defect and a pair of +½ defects.

The movie depicts the results of simulations of a transient activation of global fiber contraction in a Hydra tissue spheroid with a nematic configuration containing a +1 defect on one side of the spheroid and a pair of +½ defects at the opposite end, emulating the actin fiber organization in regenerating fragments at later stages of the regeneration process (Fig. 5C). The tissue spheroid is modeled as a deformable shell that contains an embedded nematic field that experiences a time-dependent global activation of contraction (bottom graph). The images show cell segmentation maps depicting cells colored according to the cell area strain defined as $\epsilon_c = \ln\left(A_c/A_c^{ref}\right)$, where A_c is the cell area and A_c^{ref} is the initial cell area, overlaid with lines indicating the orientation of the embedded nematic field in each cell. The spheroid is shown from a top view centered on the aster-shaped +1 defect, and a bottom view showing the pair of +½ defects. The active stress generated within each cell is aligned with the nematic field in that cell, and global activation leads to efficient mechanical strain focusing at the +1 defect site (right), and to a lesser extent at the pair of +½ defects (left).

Development • Supplementary information

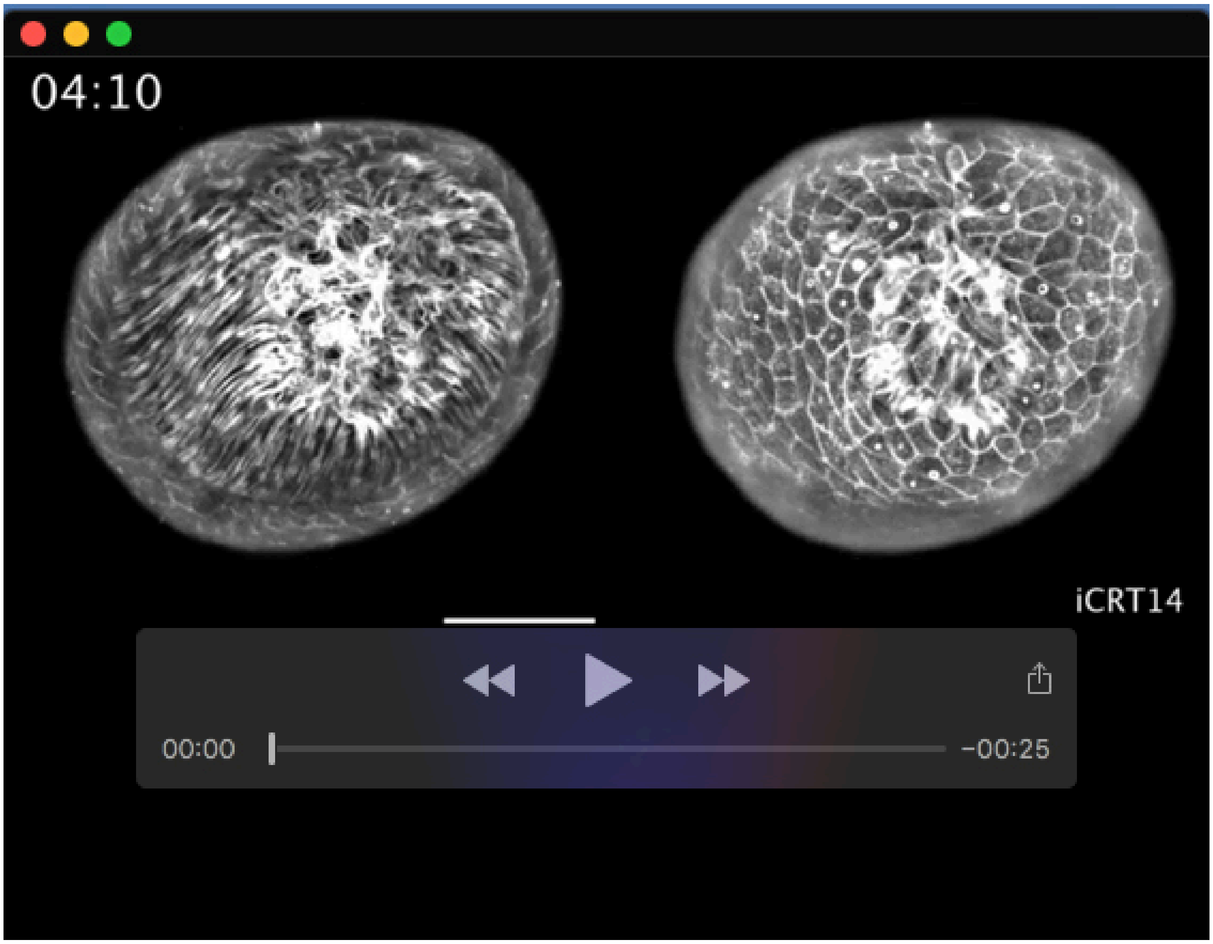

Movie 9. The effect of iCRT14 treatment on actomyosin fiber organization.

Time-lapse, spinning-disk confocal movie of a regenerating spheroid expressing Lifeact-GFP in the ectoderm, that was treated with 5 µM iCRT14 (Fig. 6B). The tissue spheroid was initially oriented with its closure region facing the objective. The actomyosin fibers do not reform in the closure region and point defects do not emerge, whereas the inherited aligned fibers visible around the closure region are maintained. The images show computationally generated projected views of the fiber organization at the ectodermal basal surface (left) and the cellular organization at the ectodermal apical surface (right). The images were centered to correct for movements of the whole tissue. The elapsed time from excision is displayed (hh:mm), and the scale bar is 100 µm.

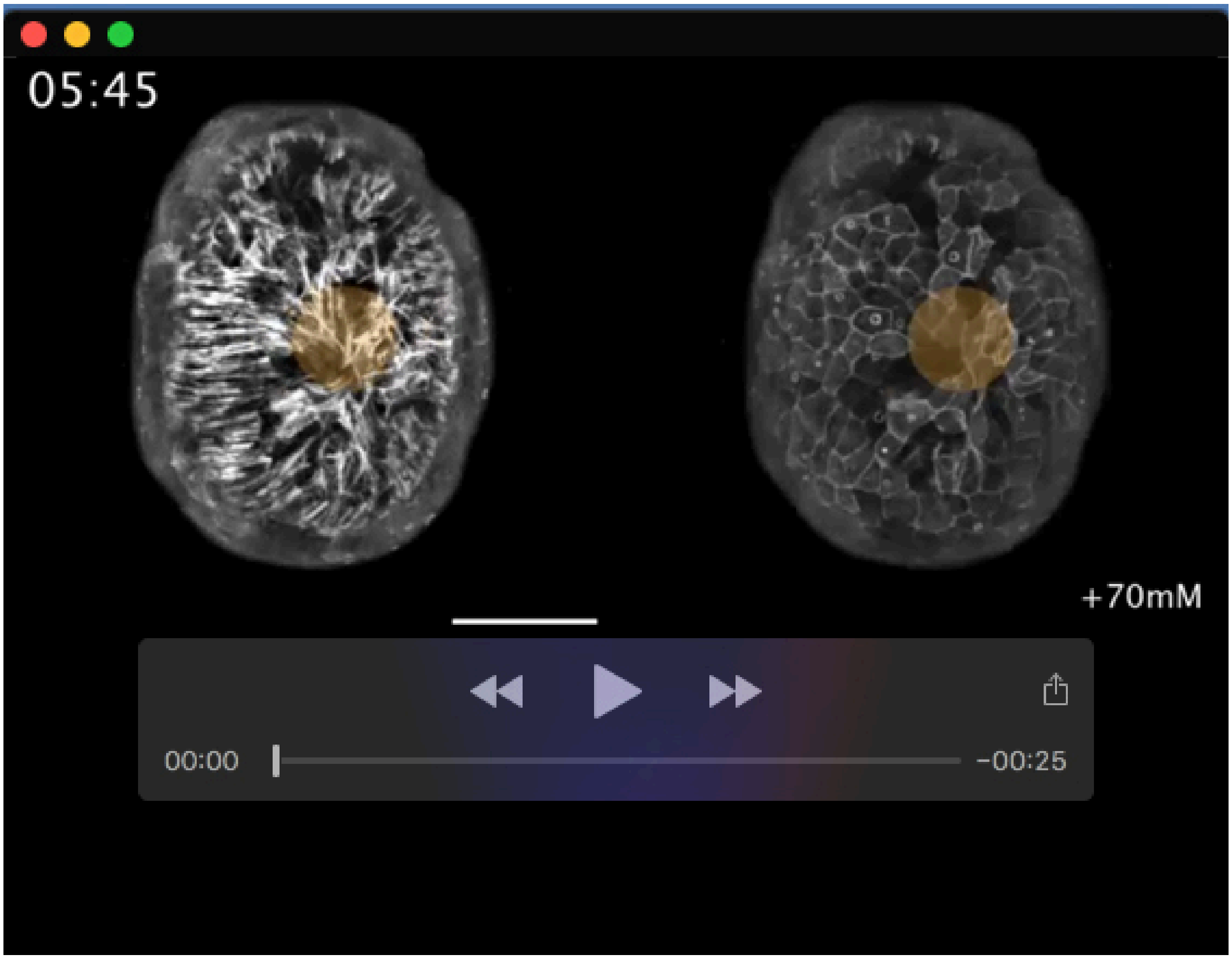

Movie 10. The effect of elevated medium osmolarity on actin fiber organization.

Time-lapse, spinning-disk confocal movie of a regenerating tissue spheroid expressing Lifeact-GFP in the ectoderm, placed in an isotonic medium containing 70 mM sucrose (Fig. 6E). The movie shows a disordered region that rapidly orders and develops a +1 defect (orange labels), that subsequently unbinds into a pair of +½ defects (cyan labels). The tissue spheroid stabilizes in a 4x +½ defect configuration and elongates along the fibers' direction but does not regenerate. The images show computationally generated projected views of the fiber organization at the ectodermal basal surface (left) and the cellular organization at the ectodermal apical surface (right). The images were centered to correct for movements of the whole tissue. The elapsed time from excision is displayed (hh:mm), and the scale bar is 100 µm.

Development • Supplementary information

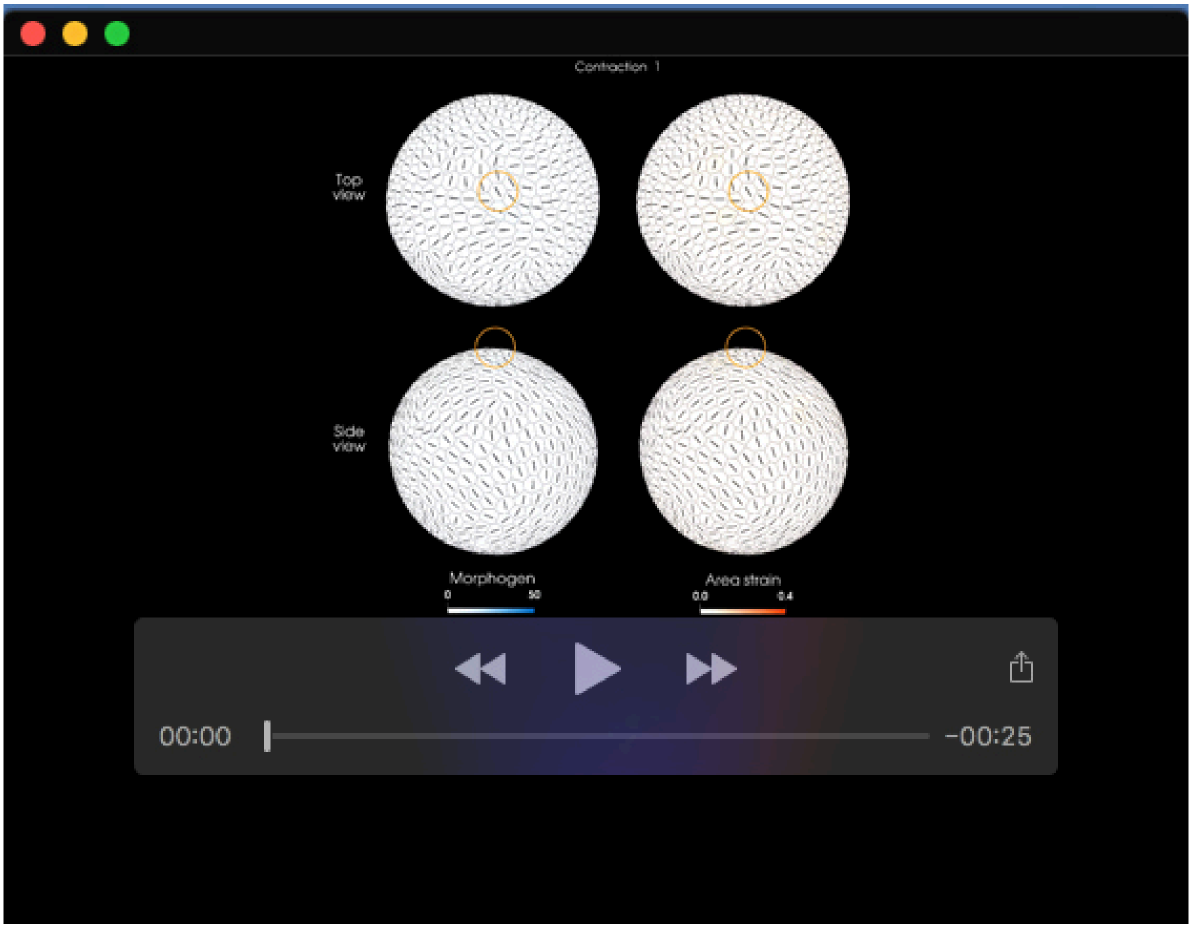

Movie 11. Simulation of Hydra tissue regeneration resulting in an asymmetric defect configuration with a +1 defect and a pair of +½ defects.

Simulation results showing a regenerating tissue spheroid initiated with a partially ordered fiber configuration and no morphogen (Fig. 7C; see supplementary Materials and Methods for details). The tissue deformations and the dynamics of the nematic and morphogen concentration fields are modeled through 30 recurring global fiber contraction events (the cycle is indicated). The spheroid is shown from a top view centered on one of the actin foci and a side view. The tissue self-organizes into a configuration with an aster-shaped +1 defect colocalized with a morphogen peak at one end of the spheroid (emulating the organizer emerging at the future head site), and a pair of +½ defects at the opposite side.

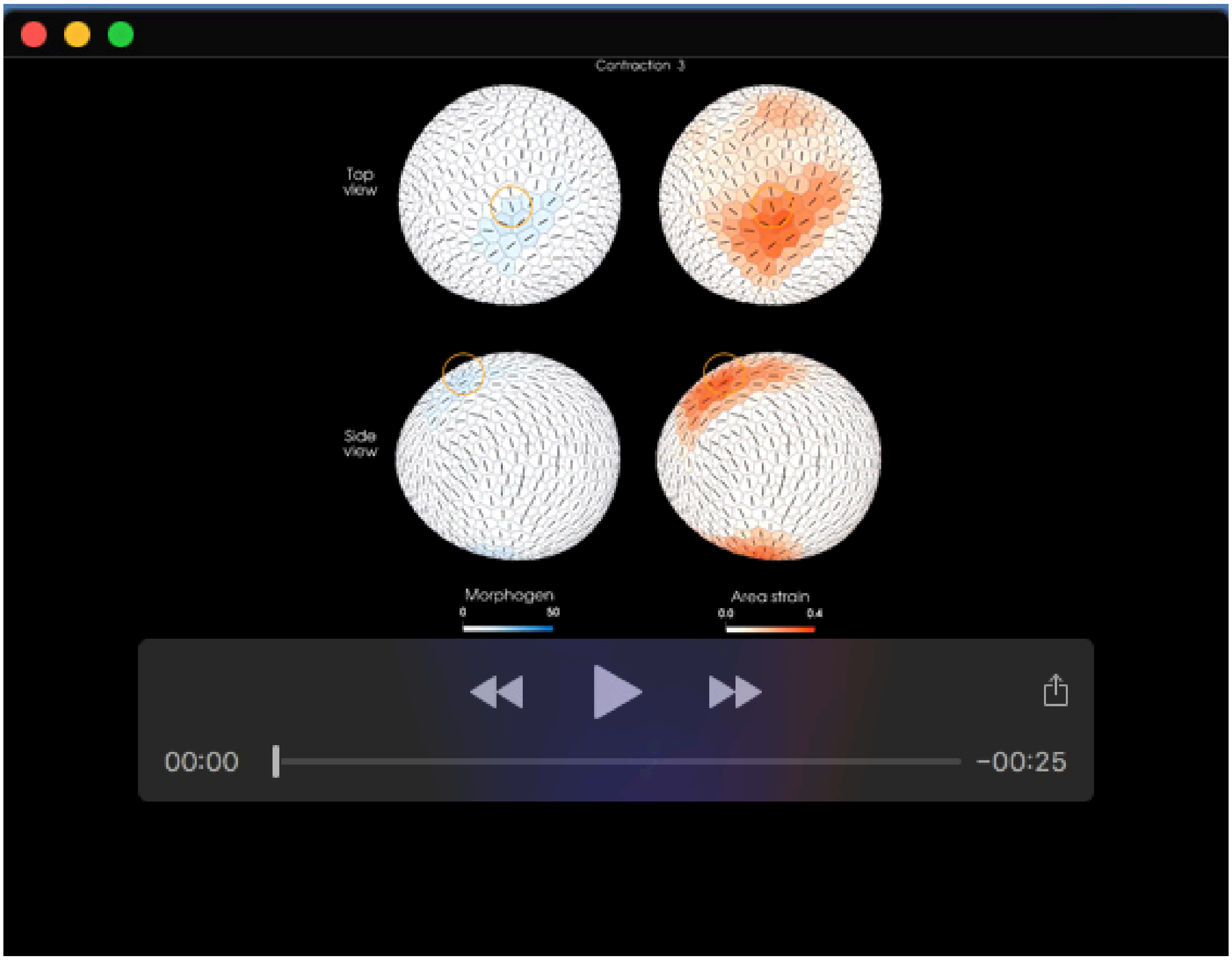

Movie 12. Simulation of Hydra tissue regeneration resulting in a symmetric defect configuration with two +1 defects.

Simulation results showing a regenerating tissue spheroid initiated with a partially ordered fiber configuration and no morphogen (Fig. 7C see supplementary Materials and Methods for details). The tissue deformations and the dynamics of the nematic and morphogen concentration fields are modeled through 30 recurring global fiber contraction events (the cycle is indicated). The spheroid is shown from a top view centered on one of the actin foci and a side view. The tissue self-organizes into a configuration with two-aster shaped +1 defects colocalized with two morphogen peaks.

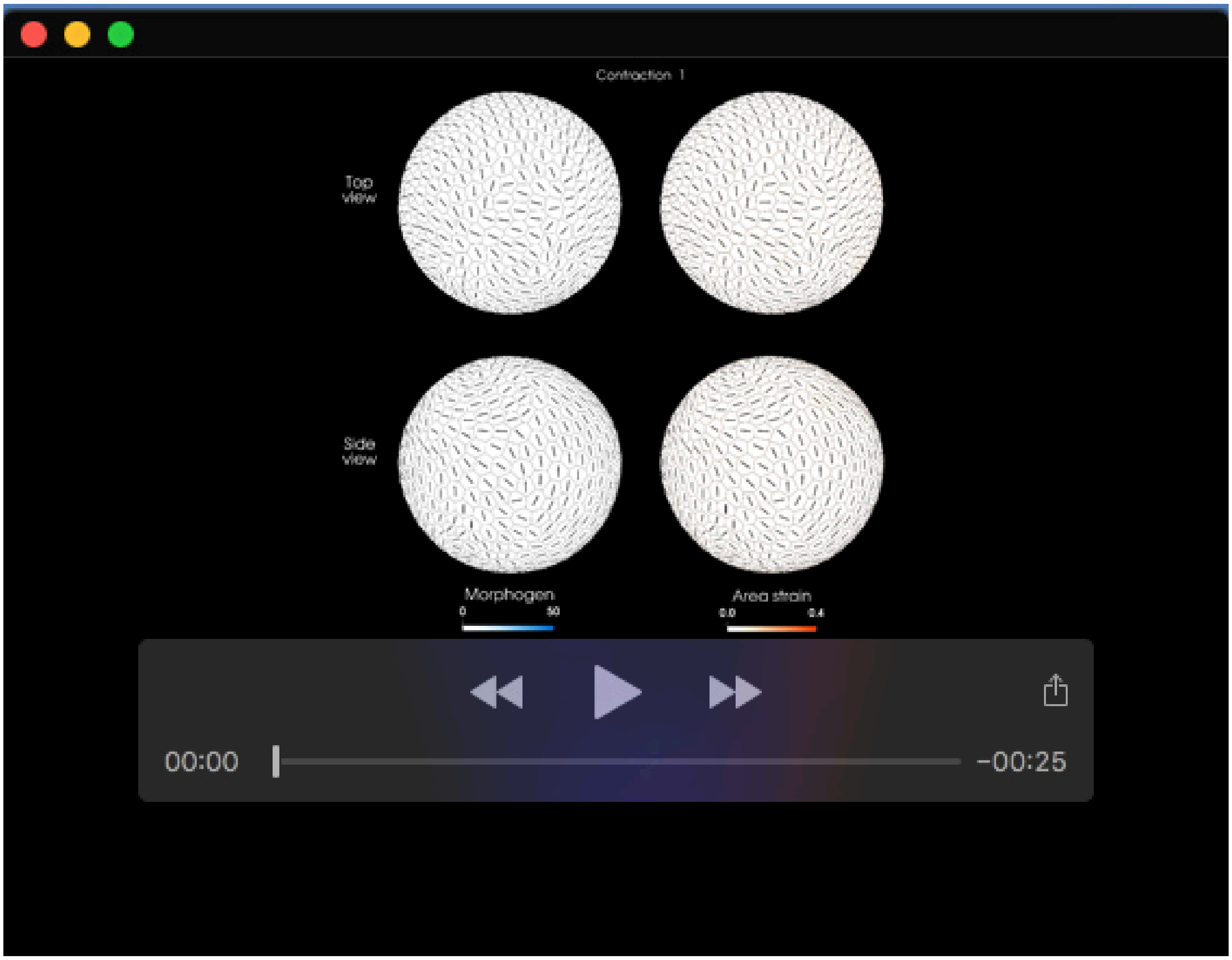

Movie 13. Simulation of Hydra tissue regeneration without morphogen.

Simulation results without coupling to a morphogen field showing a regenerating tissue spheroid initiated with a partially ordered fiber configuration (Fig. 7C; see supplementary Materials and Methods for details). The tissue deformations and the dynamics of the nematic are modeled through 30 recurring global fiber contraction events (the cycle is indicated). The spheroid is shown from a top view centered on one of the actin foci and a side view. The tissue self-organizes into a configuration with four +½ defects.

Development • Supplementary information
