# Supplementary material for: Mechanical strain focusing at topological defect sites in regenerating Hydra
Source: Development. 2025 Mar 3;152(4):DEV204514. doi: 10.1242/dev.204514 (PMC11925399; doi:10.1242/dev.204514)
Supplement: Supplementary file 1 [file develop-152-204514_review_history.pdf]

Mechanical strain focusing at topological defect sites in regenerating *Hydra*

Yonit Maroudas-Sacks, S. Suganthan, Liora Garion, Yael Ascoli-Abbina, Ariel Westfried, Noam Dori, Iris Pasvinter, Marko Popović and Kinneret Keren

DOI: 10.1242/dev.204514

Editor: Kenneth Poss

Review timeline

Original submission:	26 June 2024
Editorial decision:	10 September 2024
First revision received:	22 November 2024
Accepted:	31 December 2024

Original submission

First decision letter

MS ID#: DEVELOP/2024/203154

MS TITLE: Mechanical strain focusing at topological defect sites in regenerating Hydra

AUTHORS: Yonit Maroudas-Sacks, S. Suganthan, Liora Garion, Yael Ascoli-Abbina, Ariel Westfried, Noam Dori, Iris Pasvinter, Marko Popovic and Kinneret Keren

I have now received both referees' reports on the above manuscript, and have reached a decision. First, I apologize for this unusually long delay. The referees' comments are appended below, or you can access them online: please go to BenchPress and click on the 'Manuscripts with Decisions' queue in the Author Area.

As you will see, the referees express strong interest in your work. Reviewer #1 requests some new information and suggests some experiments as well. We would be happy to receive a revised manuscript addressing these points, which might include new experiments. Your revised paper will be re-reviewed by one or more of the original referees, and acceptance of your manuscript will depend on your addressing satisfactorily the reviewers' major concerns. Please also note that Development will normally permit only one round of major revision. If it would be helpful, you are welcome to contact us to discuss your revision in greater detail. Please send us a point-by-point response indicating your plans for addressing the referees' comments, and we will look over this and provide further guidance.

Please attend to all of the reviewers' comments and ensure that you clearly highlight all changes made in the revised manuscript. Please avoid using 'Tracked changes' in Word files as these are lost in PDF conversion. I should be grateful if you would also provide a point-by-point response detailing how you have dealt with the points raised by the reviewers in the 'Response to Reviewers' box. If you do not agree with any of their criticisms or suggestions please explain clearly why this is so.

Reviewer 1

Advance summary and potential significance to field

© 2025. Published by The Company of Biologists under the terms of the Creative Commons Attribution License (https://creativecommons.org/licenses/by/4.0/).

This manuscript investigates the relationship between tissue mechanical deformations (strain) and Wnt morphogen gradients in the establishment of apical-basal (head-tail) polarity in regenerating Hydra. The authors use an intriguing regeneration model, i.e. an excised rectangular fragment of an Hydra lateral side.
This rectangle of tissue folds into a hollow sphere, bringing the previous apical and basal sides of the tissue together, thus temporarily erasing polarity. Thereafter, the hollow sphere re-organizes, Establishes apical-basal polarity anew and eventually regenerates a new head and tail. Previous work has shown that the organization of the new head and tail depends on the orientation (nematics) of actin fibers running along the body of these regenerating animals. These fibers organize in parallel to the apical-basal axis; the curved surface of the animal causes fiber's orientation to form "topological defects", i.e. points with no defined orientation, in the presumptive head (+1 defect) and tail (2 +1/2 defects).

In this manuscript, the authors show that the Hydra tissue undergoes contractions generating tissue strain that is focused on points defined by actin orientation. Just after tissue excision and sphere formation, actin fibers are divided in an organized region, inherited from the intact animal, and a disorganized region around the previous cut sites; they find that tissue strain is focused at actin foci in this region of disorganized actin. These actin foci will later become the head and tail topological defects, where also tissue strain is found to be strongest, to the point of causing major ectodermal tissue ruptures.
The authors propose a sophisticated 3D physical model for how actin fiber organization would focus tissue strain at actin foci and topological defects.

In a second part of the manuscripts, the authors study the interplay between mechanical strain and Wnt signaling in the formation of head and tail organizers at topological defects, following up on Their characterization of tissue strain and previous work. On the experimental side, the authors show that Wnt pharmacological inhibition impairs the formation of appropriate head topological defects, but instead lead to the formation of two sets of tail-like defects. The authors propose a mathematical model in which the head Wnt morphogen gradient focalizes tissue strain by organizing actin fibers orientation nearby and, in turn, tissue strain strengthens the Wnt morphogen gradient itself. The model predicts the emergence of the appropriate head and tail topological defects organization in many instances, but can also result in other configurations depending on initial conditions. This suggests that other components are needed to obtain the appropriate head and tail configurations robustly.

I find this paper a very interesting read that provides insights on tissue nematics, mechanics and morphogen gradient establishment in regeneration. I find the manuscript technically compelling. Of note, the manuscript tackles a difficult problem, i.e. the characterization and modeling of tissue mechanical deformations in a 3D contracting and growing sphere, which the authors treat thoroughly. The closed-loop mechanism proposed in the second model is intriguing, although, as clearly acknowledged by the authors, it does not recapitulate the robust establishment of the head and tail organization. In addition, the proposed closed-feedback loop mechanism lacks experimental validation in some elements. I feel that the manuscript would benefit from a stronger connection between the first part, that is focused on tissue strain, and the second part, that is focused on the interplay between strain and morphogen gradient establishment.

Comments for the author

1)Fig. 1. I find interesting that the position of future topological defects can be predicted from the actin pattern in the initially disorganized region.
However, I did not understand how these actin foci are experimentally identified.
Does the region of disorganized actin have some low level of nematic organization that can predict the position of those foci? Otherwise, do the authors use actin intensity?
2)Fig. 3. Related to the previous comment, I think that the manuscript could benefit from experimental maps of actin fiber nematics. Is there a quantitative relationship between tissue strain and actin fiber nematics? Are these observations compatible with the model of Fig. 5?
3)Fig. 6. The authors show that actin fiber nematics is perturbed following Wnt inhibition. Is the pattern of tissue strain also perturbed? If so, is it perturbed in a way that is coherent with the formation of four +½ defects?

© 2025. Published by The Company of Biologists under the terms of the Creative Commons Attribution License (https://creativecommons.org/licenses/by/4.0/).

4)Fig. 7. As mentioned above, the model could benefit from some more experimental validation of the relationship between tissue strain and morphogen gradient establishment. I invite the authors to perform two experiments in this direction:
a)Relationship between tissue strain and morphogen gradient establishment. I suggest the authors to impair actin nematics or tissue strain and assess potential
effects on the establishment of the Wnt morphogen gradient. In this system, is there a way to re-organize actin fibers or slightly perturb actomyosin contractility?
b)Relationship between the morphogen gradient, actin nematics and tissue strain. I suggest the authors to over-express Wnt and test potential effects on actin nematics and strain around actin foci and topological defects.
5)Fig. 7 and Discussion. I suggest the authors to elaborate on what potential mechanism could provide robustness in topological defect establishment compared to those that have been included in the model.
6)Supplemental Movies. I invite the authors to indicate experimental conditions directly on the movies, even if already described in the Legends, to increase accessibility.

Reviewer 2

Advance summary and potential significance to field

This paper presents a set of impressive quantitative live imaging analyses of mechanical events during Hydra regeneration, complemented by a compelling nematic vertex model that accounts for the feedback between mechanical forces and morphogen gradients. Overall, the paper is well written, the data is intriguing, and the experimental methods and the theoretical model should be useful for the mechano-biology community working on Hydra regeneration.

Comments for the author

This is a great paper. I don't have much to add, but I found Figure S6 quite intriguing. I wonder if the authors have explored other parameters. Is the strain threshold for morphogen production the only determining factor? Is it possible to construct a 'phase diagram' and expand the discussion on this set of analyses?

In my opinion, the observed symmetry breaking is a very interesting result that deserves further exploration.

First revision

Author response to reviewers' comments

Reviewer 1

Advance Summary and Potential Significance to Field:

This manuscript investigates the relationship between tissue mechanical deformations (strain) and Wnt morphogen gradients in the establishment of apical-basal (head-tail) polarity in regenerating Hydra. The authors use an intriguing regeneration model, i.e. an excised rectangular fragment of an Hydra lateral side. This rectangle of tissue folds into a hollow sphere, bringing the previous apical and basal sides of the tissue together, thus temporarily erasing polarity. Thereafter, the hollow sphere re-organizes, establishes apical-basal polarity anew and eventually regenerates a new head and tail. Previous work has shown that the organization of the new head and tail depends on the orientation (nematics) of actin fibers running along the body of these regenerating animals. These fibers organize in parallel to the apical-basal axis; the curved surface of the animal causes fiber's orientation to form "topological defects", i.e. points with no defined orientation, in the presumptive head (+1 defect) and tail (2 +1/2 defects).

© 2025. Published by The Company of Biologists under the terms of the Creative Commons Attribution License (https://creativecommons.org/licenses/by/4.0/).

In this manuscript, the authors show that the Hydra tissue undergoes contractions generating tissue strain that is focused on points defined by actin orientation. Just after tissue excision and sphere formation, actin fibers are divided in an organized region, inherited from the intact animal, and a disorganized region around the previous cut sites; they find that tissue strain is focused at actin foci in this region of disorganized actin. These actin foci will later become the head and tail topological defects, where also tissue strain is found to be strongest, to the point of causing major ectodermal tissue ruptures. The authors propose a sophisticated 3D physical model for how actin fiber organization would focus tissue strain at actin foci and topological defects.

In a second part of the manuscripts, the authors study the interplay between mechanical strain and Wnt signaling in the formation of head and tail organizers at topological defects, following up on their characterization of tissue strain and previous work. On the experimental side, the authors show that Wnt pharmacological inhibition impairs the formation of appropriate head topological defects, but instead lead to the formation of two sets of tail-like defects. The authors propose a mathematical model in which the head Wnt morphogen gradient focalizes tissue strain by organizing actin fibers orientation nearby and, in turn, tissue strain strengthens the Wnt morphogen gradient itself. The model predicts the emergence of the appropriate head and tail topological defects organization in many instances, but can also result in other configurations depending on initial conditions. This suggests that other components are needed to obtain the appropriate head and tail configurations robustly.

I find this paper a very interesting read that provides insights on tissue nematics, mechanics and morphogen gradient establishment in regeneration. I find the manuscript technically compelling. Of note, the manuscript tackles a difficult problem, i.e. the characterization and modeling of tissue mechanical deformations in a 3D contracting and growing sphere, which the authors treat thoroughly. The closed-loop mechanism proposed in the second model is intriguing, although, as clearly acknowledged by the authors, it does not recapitulate the robust establishment of the head and tail organization. In addition, the proposed closed-feedback loop mechanism lacks experimental validation in some elements. I feel that the manuscript would benefit from a stronger connection between the first part, that is focused on tissue strain, and the second part, that is focused on the interplay between strain and morphogen gradient establishment.

Reviewer 1 Comments for the Author:

1) Fig. 1. I find interesting that the position of future topological defects can be predicted from the actin pattern in the initially disorganized region. However, I did not understand how these actin foci are experimentally identified. Does the region of disorganized actin have some low level of nematic organization that can predict the position of those foci? Otherwise, do the authors use actin intensity?

The early disordered domain mostly lacks supracellular actin fibers so the location of the foci is not determined based on local measures of the nematic organization or actin intensity. Rather, we identify the location of the foci based on the pattern of aligned fibers in the ordered domains surrounding the foci.

This is now more clearly explained at the end of paragraph 1 of the Results section (the added text is in italics): “This characteristic pattern allows us to identify two focus points of the actomyosin fiber organization, *corresponding to the centers of the disordered caps. The location of these foci is determined based on the fiber organization in the surrounding ordered regions, where the fibers form a nearly-radial pattern around each cap. The center of the caps can be approximated by extending lines along the radial fibers from the surrounding regions to their intersection point within the disordered region* (Figs. 1a,b, S1).”

2) Fig. 3. Related to the previous comment, I think that the manuscript could benefit from experimental maps of actin fiber nematics. Is there a quantitative relationship between tissue strain and actin fiber nematics? Are these observations compatible with the model of Fig. 5?

We thank the reviewer for this suggestion. We have added experimental maps of the nematic alignment of the actin fibers to Fig. 3 (overlaid on the cell area and anisotropy data n 3B,C,I,J) and

© 2025. Published by The Company of Biologists under the terms of the Creative Commons Attribution License (https://creativecommons.org/licenses/by/4.0/).

the corresponding supplementary movies (Movies 3 and 4). We have also added more details in the methods section regarding the quantification of the nematic field. Moreover, as detailed below, we have added a supplementary figure (Fig. S4) that depicts the relation between the nematic pattern and the tissue strain.

There is a quantitative relation between the tissue strain and the actin fiber nematic organization. However, this relation is not a local relation between the two fields. Rather, the strain in the elastic tissue arises from the stresses generated by the nematic actin fibers throughout the tissue. As such, the relation between the nematic field and the strain field connects the overall pattern of these two fields throughout the tissue, rather than being a local relation. We have added a supplementary figure in the revised manuscript (Fig. S4), to explicitly depict the relation between the nematic fiber organization and the tissue strain field. In particular, Fig. S4D shows that the strain pattern around a +1 defect in regenerating Hydra (data from Fig. 3), agrees with the model-predicted strain pattern around such an aster-shaped defect (simulations from Fig. 5), indicating that the observed relation between the strain field and the nematic pattern is compatible with model predictions.

This important point is also explicitly explained in the revised main text, both in the description of the experimental results in section ***Characterization of the deformation pattern during stretching events*** (describing the data in Fig. 3), and in section ***Theoretical modeling of mechanical strain focusing at actin foci*** when describing the simulations in Fig. 5.

3) Fig. 6. The authors show that actin fiber nematics is perturbed following Wnt inhibition. Is the pattern of tissue strain also perturbed? If so, is it perturbed in a way that is coherent with the formation of four +½ defects?

Following the reviewer’s comments, we have added a more detailed analysis of the tissue deformations in samples placed in a medium of high osmolarity (Fig. S6), which has been shown to inhibit the Wnt signaling pathway (Ferenc *et al.* 2021). Specifically, we observe an appreciable reduction in the extent of tissue deformation when the actin organization pattern reaches a configuration of four +½ defects. This includes fewer large deformation and rupture events (and no large ruptures). In this case, the strain pattern in both foci exhibit primarily small events, which are comparable to the events seen in the future foot region in control samples that contains a pair of +½ defects, and smaller than the typical events observed in the +1 defect at the future head side. This is presented in the revised manuscript (Fig. S6A-C,E-G). We also present a comparison of the defect configuration over time in normal media and high osmolarity media (Fig. S6D,H).

4) Fig. 7. As mentioned above, the model could benefit from some more experimental validation of the relationship between tissue strain and morphogen gradient establishment. I invite the authors to perform two experiments in this direction:
a)Relationship between tissue strain and morphogen gradient establishment. I suggest the authors to impair actin nematics or tissue strain and assess potential effects on the establishment of the Wnt morphogen gradient. In this system, is there a way to re-organize actin fibers or slightly perturb actomyosin contractility?

While it is possible to perturb the organization and contraction of the actomyosin fibers, these experiments are unfortunately limited in their ability to shed light on the relation between tissue strain and morphogen distribution. There is currently no way to measure the Wnt morphogen gradient in live animals. The published studies of the spatial distribution of the Wnt signaling pathway are based on *in situ* hybridization in fixed animals. This provides information on where Wnt is expressed, but not on the gradient of the morphogen itself (i.e. protein levels) and is limited to discrete time-points in different fixed samples, which is problematic given the large inherent variability in regeneration time across different samples in Hydra. Furthermore, it is not possible in these studies to correlate the *in situ* hybridization Wnt signals with the actin fiber organization. As such, we do not have a good readout of the Wnt morphogen gradient in Hydra, beyond studying the regeneration outcome (i.e. where a head eventually forms in the regenerating Hydra).

Perturbations of myosin activity (e.g. with blebbistatin) or actin dynamics (by cytochalasin or latrunculin) block regeneration but the tissue also disintegrates over time (see for example our

© 2025. Published by The Company of Biologists under the terms of the Creative Commons Attribution License (https://creativecommons.org/licenses/by/4.0/).

published work, Livshits *et al. Cell Reports* 2017). Given the central role of the actomyosin cytoskeleton to basic physiological functions, these results do not provide much insight into the relation between Wnt signaling and the actin fiber organization. As such, while we agree with the reviewer that the relation between tissue strain and morphogen gradient establishment is a central point for validating our model, answering it is beyond the scope of the current manuscript.

We should note, that changing the fiber distribution in regenerating tissue spheroids by modulating the excised tissue shape and/or applying constraints on the tissue does provide support for the proposed relation between the defect configuration and the morphogen pattern as reflected by the presence or absence of extra regenerated heads. Specifically, perturbations which induce more defects early on, result in higher probability for the formation of multiaxial animals. This includes regenerating open rings (Livshits *et al. Cell Reports* 2017, Maroudas-Sacks *et al., Nature Physics* 2021) and tissue spheroids confined in a frustrated orientation in narrow channels (Maroudas-Sacks *et al. PRX Life,* 2024*)*. Conversely, in tissue tori where the nematic actin fibers are fully aligned and no nematic defects are present, there is a reduced probability for forming a head (Ravichandran *et al., BioRxiv* 2024).

b) Relationship between the morphogen gradient, actin nematics and tissue strain. I suggest the authors to over-express Wnt and test potential effects on actin nematics and strain around actin foci and topological defects.

Following the reviewer's comments, we have done additional experiments and followed the actin fiber dynamics under conditions of global Wnt overexpression. This is done using transgenic Hydra expressing lifeact-GFP (to visualize actin fiber dynamics) and applying pharmacological inhibitors to upregulate the Wnt signaling pathway. We use Alsterpaullone, which has been shown to inhibit the glycogen synthase kinase-3ßcomplex and lead to elevated ß-catenin levels and upregulation of the Wnt pathway (Broun *et al., Development* 2005; Reddy *et al., Current Biology* 2019; Fig. R1A). This perturbation leads to regeneration of abnormal Hydra with multiple tentacles and heads, which is correlated with the formation of excess defects (Fig. R1B). We can emulate this behavior in our model, by introducing a basal level of stochastic morphogen production in all cells (Fig. R1C). While these additional results are consistent with the proposed mechanism, we do not feel they are sufficient to validate the proposed mechanism, and hence prefer not to include them in the revised manuscript. We plan to study in more detail the relation between the Wnt signaling pathway and actin fiber organization in future work. In particular, we believe that combining such global perturbations with local perturbations should be more informative regarding the relation between morphogen gradients and actin nematics. However, these local perturbations require developing the methodology to apply the drug locally in the highly dynamics regenerating tissue which is not straightforward, and are hence beyond the scope of the current manuscript.

NOTE: We have removed unpublished data that had been provided for the referees in confidence.

Figure R1. The influence of Alsterpaullone-treatment on actin fiber organization and regeneration outcome A) *In situ* hybridization of HyWnt3 in mature control Hydra (left) and in Hydra treated with 5μM Alsterpaullone for 24 hours (right; images adapted from Reddy *et al., Current Biology* 2019). In control Hydra, HyWnt3 is expressed only at the organizer site at the tip of the mouth, whereas in Alsterpaullone-treated Hydra Wnt expression is present throughout the body column. B) Spinning disk images from a movie of a regenerating Hydra tissue excised from an animal treated with 5 μM Alsterpaullone for 24 hours prior to excision. Images of the actin fiber organization at an early (top) and later (bottom) time point are shown. The tissue develops excess defects, which result in an abnormal morphology with multiple protrusive structures. C) Outcome of model simulations with overexpression of morphogen, implemented by adding a basal level of strain-independent morphogen production. In each cell, the basal level of morphogen production is taken from a Gamma distribution with mean=std=0.05. All other model parameters, including the strain-dependent morphogen production, are identical to those used in Fig. 7. The tissue develops excess defects and multiple morphogen peaks.

5) Fig. 7 and Discussion. I suggest the authors to elaborate on what potential mechanism could provide robustness in topological defect establishment compared to those that have been included in the model.

© 2025. Published by The Company of Biologists under the terms of the Creative Commons Attribution License (https://creativecommons.org/licenses/by/4.0/).

As the reviewer notes, the simulation results (Fig. S7) are more variable in terms of the outcome defect configuration compared to experiments where we find that regenerating fragments develop a +1 defect and a pair of +1/2 defects in a robust manner. We have previously shown that this polar defect configuration reflects a strong memory of tissue polarity in the excised tissue originating from the parent animal (Shani-Zerbib *et al.*, *Genes* 2022). It is still unclear what mediates this memory of polarity, which is why this feature has not been included in the current model. We believe that bias in the initial conditions (i.e. the bias between the initially head and foot-facing sides of the excised tissue) helps promote the robust establishment of a polar defect configuration in regenerating fragments, which later develops into a mature hydra with a polar body plan. This important point is now explained in the discussion of the revised manuscript.

6) Supplemental Movies. I invite the authors to indicate experimental conditions directly on the movies, even if already described in the Legends, to increase accessibility.

We have added annotations of the experimental conditions in the different perturbations on the movies as requested.

Reviewer 2

Advance Summary and Potential Significance to Field:
This paper presents a set of impressive quantitative live imaging analyses of mechanical events during Hydra regeneration, complemented by a compelling nematic vertex model that accounts for the feedback between mechanical forces and morphogen gradients. Overall, the paper is well written, the data is intriguing, and the experimental methods and the theoretical model should be useful for the mechano-biology community working on Hydra regeneration.

Reviewer 2 Comments for the Author:
This is a great paper. I don't have much to add, but I found Figure S6 quite intriguing. I wonder if the authors have explored other parameters. Is the strain threshold for morphogen production the only determining factor? Is it possible to construct a 'phase diagram' and expand the discussion on this set of analyses? In my opinion, the observed symmetry breaking is a very interesting result that deserves further exploration.

We thank the reviewer for the appreciation of our work.

The proposed model has two main determining features- the strain-dependent morphogen production and the morphogen gradient-dependent fiber alignment. The strain threshold for morphogen production (ϵ_{th}) characterizes the former, whereas the nematic-morphogen coupling strength (a) characterizes the latter. Following the reviewer's comment, we have expanded our analysis and now consider the behavior of the model as a function of both the morphogen production threshold ϵ_{th} and the nematic-morphogen coupling strength α.

We find that increasing the production threshold ϵ_{th} leads to more prevalent +1/2 defects, which is easy to understand as the reduced morphogen production in the system makes it harder to stabilize +1 defects. Furthermore, for each value of ϵ_{th}, increasing α leads to an increasing number of +1 defects, which can be understood to be due to the easier nucleation and stabilization of +1 defects in the presence of stronger nematic coupling to the morphogen gradient. The frequency of samples having excess defects, including negative -1/2 defects, however, changes non-monotonically with α. At low values of α we sometimes find long-lasting (i.e. longer than 30 contractions) -1/2 defects, since the resolution of excess defects through nearest neighbor coupling is slow at long distances. At intermediate values of α, these -1/2 defects do not form since coupling to the morphogen diffusing radially from +1 defect regions orients the nematic pattern on a large part of the corresponding hemisphere. Finally, at high values of α, more complex patterns form with additional +1 and -1/2 defects. The parameter choice we use in the main text falls in the intermediate regime, where the strain threshold ϵ_{th} is sufficiently low to allow for production of morphogen, and the coupling to the morphogen is sufficient to induce +1 defect(s) and enhance nematic ordering, but not too high as to frequently induce excess defects in the actin organization.

© 2025. Published by The Company of Biologists under the terms of the Creative Commons Attribution License (https://creativecommons.org/licenses/by/4.0/).

The revised manuscript now contains a diagram summarizing the regeneration outcomes from model simulations done in the space spanned by these two parameters (revised Fig. S7), which are discussed in the results section and explained in more detail in the Supplementary Text.

Regarding symmetry breaking. We agree with the reviewer that this is an interesting feature that deserves further analysis. Following the reviewer's comments, we have performed additional simulations to better characterize the origin of the asymmetric solutions obtained (i.e. solutions with a +1 defect on one side and a pair of +1/2 defects on the other side). The type of defect configuration that stabilizes at actin foci, following the induction of nematic order, depends on the strain threshold for morphogen production; the system favors the formation of +1 defects at low strain threshold values, whereas at higher strain threshold values, only pairs of +1/2 defects develop (Fig. S7). To study the origin of the asymmetric solutions, we examined the simulation results at intermediate strain threshold values, where both solutions (i.e. +1 defects and pairs of +1/2 defects) are possible. We characterized the statistics of the simulation outcome for 100 different realizations of the initial conditions for each set of model parameters. Specifically, we examined whether the formation of asymmetric defect configurations reflects the joint probability of the two foci to independently develop into a +1 defect and a pair of +1/2 defects, respectively, or rather an inherent symmetry breaking in which the formation of a +1 defect on one side influences the probability of forming another +1 defect at the opposite side.

We find that the statistics of outcome defect configurations in the simulations are consistent with assuming that each actin focus develops essentially independently into a +1 defect or a pair of +1/2 defects, with a strain threshold-dependent probability. This is now explained in the revised manuscript. While the elastic coupling between the two foci region could potentially generate an effective interaction between the two foci that could favor an asymmetric solution (i.e. stretching at one focus would tend to contract the tissue at the opposite end), in the current model this effect is negligible. Further work, which goes beyond the scope of the current manuscript, will be needed to characterize the potential for spontaneous symmetry breaking in the model and understand how this relates to the inherent polarity memory that is evident in regenerating Hydra.

Second decision letter

MS ID#: dev.204514R1

MS TITLE: Mechanical strain focusing at topological defect sites in regenerating Hydra

AUTHORS: Yonit Maroudas-Sacks, S. Suganthan, Liora Garion, Yael Ascoli-Abbina, Ariel Westfried, Noam Dori, Iris Pasvinter, Marko Popovic and Kinneret Keren

I apologize for the delays. I am happy to tell you that your manuscript has been accepted for publication in Development, pending our standard publication integrity checks.

Comments from the Reviewers:

Reviewer 1: In this revised manuscript, the authors have provided additional clarifications, analysis and experimental insights, thus addressing satisfactorily the points that I had raised in my previous review. In particular, the authors have clarified what aspects of the relationships between tissue strain, nematics and Wnt morphogen gradient were validated experimentally and directly, indirectly or through mathematical modeling.

© 2025. Published by The Company of Biologists under the terms of the Creative Commons Attribution License (https://creativecommons.org/licenses/by/4.0/).
